# A novel sphingolipid-TORC1 pathway critically promotes postembryonic development in *Caenorhabditis elegans*

**DOI:** 10.7554/eLife.00429

**Published:** 2013-05-21

**Authors:** Huanhu Zhu, Huali Shen, Aileen K Sewell, Marina Kniazeva, Min Han

**Affiliations:** 1 Howard Hughes Medical Institute, University of Colorado, Boulder, Boulder, United States; 2 Department of Molecular, Cellular and Developmental Biology, University of Colorado, Boulder, Boulder, United States; 3 Department of Chemistry and Institute of Biomedical Sciences, Fudan University, Shanghai, China; University of Cambridge, United Kingdom

**Keywords:** branched-chain fatty acid, growth arrest, nutrient sensing, NPRL, glucosylceramide, target of rapamycin, C. elegans

## Abstract

Regulation of animal development in response to nutritional cues is an intensely studied problem related to disease and aging. While extensive studies indicated roles of the Target of Rapamycin (TOR) in sensing certain nutrients for controlling growth and metabolism, the roles of fatty acids and lipids in TOR-involved nutrient/food responses are obscure. *Caenorhabditis elegans* halts postembryonic growth and development shortly after hatching in response to monomethyl branched-chain fatty acid (mmBCFA) deficiency. Here, we report that an mmBCFA-derived sphingolipid, d17iso-glucosylceramide, is a critical metabolite in regulating growth and development. Further analysis indicated that this lipid function is mediated by TORC1 and antagonized by the NPRL-2/3 complex in the intestine. Strikingly, the essential lipid function is bypassed by activating TORC1 or inhibiting NPRL-2/3. Our findings uncover a novel lipid-TORC1 signaling pathway that coordinates nutrient and metabolic status with growth and development, advancing our understanding of the physiological roles of mmBCFAs, ceramides, and TOR.

**DOI:** http://dx.doi.org/10.7554/eLife.00429.001

## eLife digest

Animals require nutrients, including carbohydrates, lipids, and amino acids, for development and growth, and to maintain the normal functioning of cells. However, in most natural environments, the availability of food tends to fluctuate. Some animals have therefore acquired the ability to dramatically reduce their metabolic activity, and thus their energy and nutrient needs to survive fasting conditions.

*Caenorhabditis elegans* is a transparent nematode worm that is used extensively as a model organism. When *C. elegans* larvae hatch in a food-free environment, they enter a quiescent state in which they suspend growth and cell division to conserve energy. However, the mechanisms that underlie this ability are not fully understood.

Here, Zhu et al. reveal that a type of lipid called a sphingolipid is required for *C. elegans* larvae to begin postembryonic development. When this lipid is absent in the environment and not synthesized internally, the larvae remain in a state of arrested development, which can be overcome by resupplying the lipid. Zhu et al. show that the lipid acts through a signaling pathway involving an enzyme complex called TORC1 and that the effect of the lipid can be blocked by another protein complex called NPRL-2/3. TORC1 is well known for its role in sensing amino acids and growth factors, but this is the first time that it has been shown to be involved in detecting lipids. Strikingly, Zhu et al. also show that, in the absence of the lipid, postembryonic growth and development can be initiated by activating TORC1 or inhibiting NPRL-2/3.

The work of Zhu et al. thus reveals a novel regulatory function of a specific fatty acid and sphingolipid variant that is used by *C. elegans* to coordinate its growth and development with its metabolic status or the availability of nutrients. Since all components of the pathway are conserved in mammals, the results could help to improve our understanding of how caloric restriction influences human health and aging.

**DOI:** http://dx.doi.org/10.7554/eLife.00429.002

## Introduction

Regulation of animal growth and development in response to nutritional cues is an intensely studied problem (Hietakangas and Cohen, 2009; Zoncu et al., 2011). In animals, nutrient signals are perceived in specialized tissues and are then communicated to all other tissues to coordinate growth and development. The target of rapamycin (TOR) complexes (TORC1 and TORC2) are known to function in sensing various nutrient signals (Ma and Blenis, 2009; Laplante and Sabatini, 2012; Zoncu et al., 2012), and their roles are connected to growth, metabolism, stress responses, and cancers (Hansen et al., 2008; He and Klionsky, 2009; Howell and Manning, 2011). While amino acids, energy, and growth factors have been described as nutrient inputs to TOR complexes, roles of lipid molecules as signals to these systems in controlling postembryonic growth and development were not known. Clearly, it is also essential to use whole-animal models to investigate how different signaling systems in different tissues interact to specify decisions regarding postembryonic development and behaviors.

In *Caenorhabditis elegans*, the first larval stage has been established as a model system for the study of animal growth and development in response to food availability. When hatched in food-free surroundings, this nematode enters a quiescent state, termed L1 diapause (Johnson et al., 1984; Munoz and Riddle, 2003; Baugh and Sternberg, 2006). An insulin/IGF-1 receptor signaling (IIS) pathway plays a critical role in the induction of L1 diapause and survival of the developmentally arrested animals (Gems et al., 1998; Baugh and Sternberg, 2006; Kniazeva et al., 2008; Lee and Ashrafi, 2008; Jones et al., 2009; Soukas et al., 2009). Moreover, TOR complexes have also been indicated to play prominent roles in regulating postembryonic growth and lifespan in *C. elegans* (Vellai et al., 2003; Syntichaki et al., 2007; Hansen et al., 2008; Honjoh et al., 2009; Lucanic et al., 2011).

Monomethyl branched-chain fatty acids (mmBCFAs) are widely present in bacteria, plants, and animals, including humans (Nicolaides and Ray, 1965; Ran-Ressler et al., 2008). In mammals, mmBCFAs are derived from branched-chain amino acids (BCAAs) (Morii and Kaneda, 1982; Oku et al., 1994), although the remainder of the de novo pathway has not been delineated. The physiological roles of these FA variants are essentially unknown, even though they were found to be present at very high levels in certain tissues (Nicolaides and Ray, 1965; Ran-Ressler et al., 2008). In *C. elegans*, readily detectable mmBCFAs C15ISO and C17ISO are synthesized from the BCAA leucine (Kniazeva et al., 2004). The key enzymes in this de novo synthesis pathway, including the branched-chain ketoacid dehydrogenase complex (BCKDC), an FA elongase (ELO-5), and an acyl CoA synthetase (ACS-1), are evolutionarily conserved (Kniazeva et al., 2004, 2008).

We have previously shown that newly hatched *C. elegans* that are deficient for mmBCFAs cannot initiate postembryonic growth and development, and instead enter L1 diapause. Further genetic analysis suggested that this developmental arrest is independent of the IIS pathway (Kniazeva et al., 2004, 2008). It was not clear whether the essential roles of mmBCFAs and their derived lipids were due to structural requirements for animal development, as was suggested by other studies, or due to regulatory functions specific to cellular signaling processes. Testing these hypotheses using model organisms is highly significant because roles of fatty acids and lipids function as nutrient signals for postembryonic development are in general poorly understood.

In this study, we discovered that (1) an mmBCFA-derived glucosylceramide (d17iso-GlcCer) mediates the function of mmBCFAs in promoting postembryonic growth and development and (2) d17iso-GlcCer acts through a signaling system that includes the NPRL-2/NPRL-3 protein complex (negative factor) and TORC1 (positive factor) to promote postembryonic development.

## Results

### A sphingolipid composed of a mmBCFA-derived long chain base mediates the essential role of mmBCFAs in the initiation of postembryonic growth and development

*elo-5* loss-of-function mutants (termed *elo-5(−)* hereafter) are deficient for mmBCFAs and are developmentally arrested at the early L1 stage. This phenotype is completely rescued by dietary supplementation of mmBCFAs (Kniazeva et al., 2004, 2008). We found that in mmBCFA-deficient *elo-5(−)* larvae, the amount of mmBCFA-containing sphingolipids were dramatically reduced and thus asked if this mmBCFA function is mediated by a sphingolipid.

**Figure 1.**
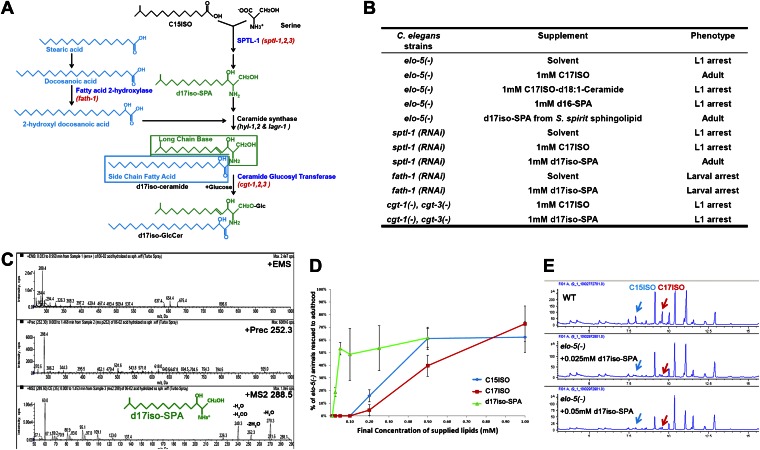
Iso-branched d17iso-sphinganine rescues elo-5(-) L1 arrest. (**A**) Sphingolipid biogenesis pathway in *C. elegans*, including the catalytic enzymes (blue) and corresponding genes (red) used in this study. Molecular structures in green and in light blue indicate the long chain base and side chain fatty acid, respectively. (**B**) Summary of growth phenotype of various mutants with indicated lipid supplementations. *elo-5(gk208)*, *cgt-1(tm1027)*, and *cgt-3(tm504)* were the alleles of the (−) mutants. LCB: long chain base; SPA: sphinganine. See Supplementary file 1 for numerical data or more detailed description of the phenotypes. (**C**) Mass spectrometry of iso-branched d17iso-sphinganine (d17iso-SPA) isolated from the bacteria *S. spiritivorum*. The major peak (*m/z* = 288.4) corresponds to d17iso-SPA in each panel. The lower panel shows fragmentation of d17iso-SPA by MS-MS scan. The fragment peaks are labeled with the name of lost residues. (**D**) d17iso-SPA rescues *elo*-5*(−)* animals more efficiently than C15ISO and C17ISO. Error bar, SD. (**E**) Gas chromatography of methyl-esterified fatty acid extracts from rescued *elo*-5*(−)* animals fed with d17iso-SPA. Low concentration of d17iso-SPA (second and third panels) supplement did not restore C15iso or C17iso fatty acid levels. **DOI:** http://dx.doi.org/10.7554/eLife.00429.003

Sphingolipids are a class of well-known bioactive lipids (Hannun and Obeid, 2008) that are composed of an aliphatic amino alcohol backbone called the sphingoid base or long chain base (LCB), and usually an N-acylated fatty acid (FA) side chain (Figure 1A). In *C. elegans*, both the LCB and the FA side chain may derive from mmBCFAs, such as C15ISO and C17ISO (Chitwood et al., 1995; Gerdt et al., 1997; Figure 1A). In this study, a sphingolipid with a mmBCFA-derived LCB is termed d17iso-sphingolipid, whereas a sphingolipid with a C17ISO-derived FA side chain and a straight LCB is termed c17iso-sphingolipid. The ‘d’ and ‘c’ refer to carbon atoms on the LCB and FA chain, respectively.

We then examined if dietary supplementation with either of these two types of sphingolipids could substitute for mmBCFAs to rescue *elo-5(−)* animals from L1 arrest. Because the d17iso-LCB was not commercially available, we purified d17iso-sphinganine (d17iso-SPA; Figure 1A) from a d17iso-sphingolipid producing bacteria *S. spiritivorum* (Yabuuchi et al., 1983) using mass spectrometry analysis. This purified d17iso-SPA fraction, but not a chemically synthesized c17iso-sphingolipid with a straight-chain d18:1-LCB or a straight-chain LCB d16-SPA, was found to be sufficient to overcome *elo-5(−)* L1 arrest (Figure 1B,C; Supplementary file 1). Additionally, efficient rescue was also achieved with a custom synthesized d17iso-SPA (Figure 1D). Therefore, d17iso-sphingolipid is sufficient to promote the postembryonic growth and development of mmBCFA-deficient animals.

We then carried out further tests to confirm that this rescue effect of d17iso-SPA supplementation resulted from the function of this metabolite itself, but not from its catabolic conversion back to C15ISO or C17ISO. First, we fed *elo-5(−)* larvae with various quantities of C15ISO, C17ISO, or d17iso-SPA. We observed that d17iso-SPA suppressed the L1 arrest at a much lower concentration than C15ISO or C17ISO (Figure 1D). Second, we observed that the low concentrations of d17iso-SPA suppressed the L1 arrest without restoring mmBCFA levels (Figure 1E). Finally, we found that supplementation with d17iso-SPA, but not mmBCFAs, also suppressed the L1 arrest phenotype caused by feeding RNAi of *sptl-1*, a gene encoding a serine palmitoyltransferase homolog in *C. elegans* required for d17iso-SPA biosynthesis (Figure 1A,B; Seamen et al., 2009). We thus conclude that mmBCFAs promote postembryonic development through d17iso-SPA.

This growth-promoting function of d17iso-SPA may be mediated by a more complex sphingolipid derived from it. Because it is technically difficult to purify or synthesize sphingolipids of more complex structure (e.g., glucosylceramide [GlcCer]) and directly test their effect by dietary supplementation, we examined additional enzymes involved in sphingolipid synthesis. Specifically, *fath-1(C25A1.5)* encodes a homolog of mammalian fatty acid 2-hydrolase (FA2H) responsible for the synthesis of the FA side chain of ceramides, whereas *cgt-1* and *cgt-3* encode two ceramide glucosyltransferases that glucosylate ceramides to GlcCer (Figure 1A). Both *fath-1(RNAi)-*treated animals (Figure 1B; Supplementary file 1) and the *cgt-1(−);cgt-3(−)* double mutant (Marza et al., 2009) cause larval arrest. We found the arrest under either condition could not be overcome by feeding d17iso-SPA (Figure 1B; Supplementary file 1). Disrupting the function of glycosyltransferases that further modify GlcCer does not cause larval arrest (Griffitts et al., 2003). These results suggest that d17iso-glucosylceramide (d17iso-GlcCer) (Figure 1A) may be the lipid molecule that mediates the role of mmBCFAs and d17iso-SPA in promoting postembryonic growth and development in *C. elegans*.

### Mutation *ku540* permits mmBCFA-depleted *C. elegans* to bypass L1 arrest and propagate continuously

**Figure 2.**
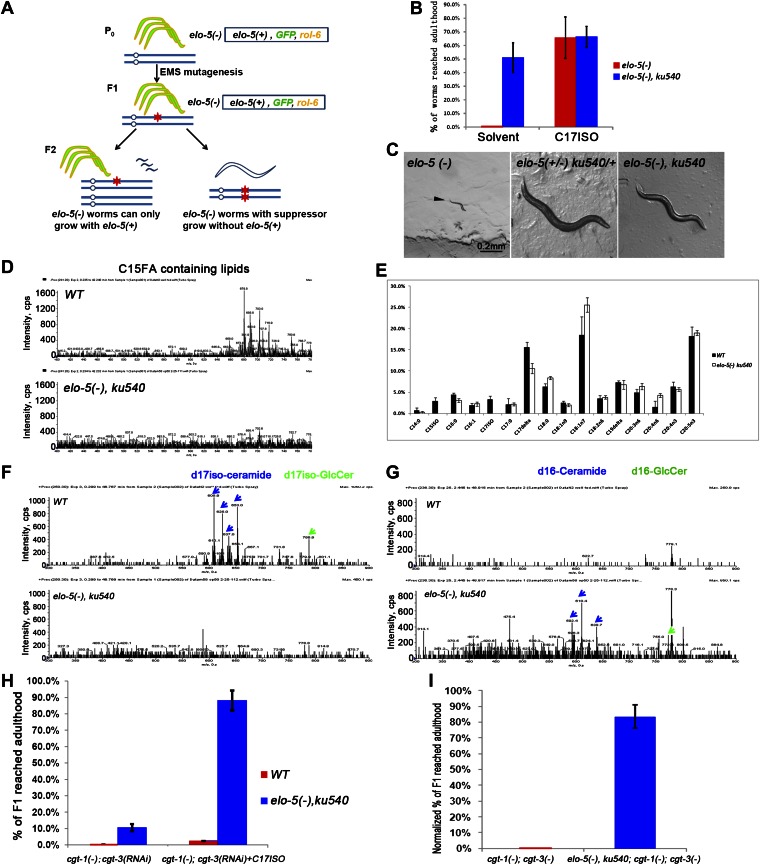
*ku540* mutant suppresses L1 arrest of mmBCFA and sphingolipid biosynthetic mutants without restoring the levels of mmBCFAs. (**A**) Screening strategy to isolate *elo*-5*(−)* suppressors. Green-colored *C. elegans* carry the extrachromosomal array containing copies of the *elo-5(+), sur-5-gfp*, and *rol-6(dn)* genes. (**B**) Percentages of animals reaching adulthood (bypass L1 arrest) when fed with/without C17ISO supplement. *elo-5(−);ku540* mutant animals reach adulthood without C17ISO supplement. Error bar, SD. (**C**) Images showing that *elo*-*5(−)* animals arrested at L1 and *elo-5(−);ku540* animals reached adulthood. (**D**) Mass spectrometry by precursor scan *m/z* = −241.2 showing no detectable C15FA-containing lipids in *elo-5(−);ku540* animals. Numeric data for the levels of PA and PE are shown in Figure 2—figure supplement 1A. (**E**) Bar graph of relative FA composition by gas chromatography (GC) of methyl-esterified fatty acid extracts. These data indicate no detectable C15ISO or C17ISO was restored in *elo-5(−) ku540* animals. GC graph is shown in Figure 2—figure supplement 1B. (**F**) and (**G**) Mass spectrometry of d17iso-ceramide–containing lipids by precursor scan *m/z* = +250.3, and d16-ceramide–containing lipids by precursor scan m/z = +236.3 (**F**). d17iso-ceramides and d17iso-glucosylceramides are detectable in wild-type but not *elo-5(−);ku540* animals (**G**). In contrast, d16-ceramides and d16-glucosylceramides are present in *elo-5(−);ku540,* but not wild-type, animals. Numeric data for relevant peaks are shown in Figure 2—figure supplement 1C,D. (**H**) and (**I**) Percentages of animals of the indicated genotypes and treatment that reached adulthood. Error bar, SD. (**H**) The addition of *ku540* dramatically suppressed the L1 arrest phenotype of *cgt-1(−);cgt-3(RNAi)* with and without the C17ISO supplement. The inclusion of *elo*-5*(−)* was due to its close linkage with *ku540*. When C17ISO was added to remove the negative effect of *elo*-5*(−),* about 90% of *elo-5(−);ku540; cgt-1(−);cgt-3(RNAi)* animals reached adulthood. C17ISO itself does not rescue the phenotype. (**I**). *ku540* suppressed the L1 arrest phenotype the *cgt-1(−);cgt-3 (−)* double mutants (83.4%, n = 126). In this particular test, *elo-5(−) ku540;cgt-1(−);cgt-3(−)* homozygous animals were the progeny of *elo-5(−/+) ku540(−/+);cgt-1(−/+);cgt-3(−)* heterozygous mothers, and the presented data were obtained after normalizing against heterozygous populations (see ‘Materials and methods’). In all the other tests shown in Figure 2, *elo-5(−) ku540* homozygous animals were the progeny of homozygous mothers. **DOI:** http://dx.doi.org/10.7554/eLife.00429.004

d17iso-sphingolipid may play either a structural or regulatory role for the initiation of postembryonic development. If it is the latter, the requirement of d17iso-sphingolipid for postembryonic development might be bypassed by genetically activating the downstream regulatory pathway. To test this possibility and identify the mechanism, we performed a genetic screen, modified from a previous screen (Seamen et al., 2009), for mutations that could suppress the L1 arrest of *elo-5(−)* mutants (Figure 2A). Among four suppressors identified in this screen, and the *tat-2* mutations from a previous *elo-5(−)* suppressor screen (Seamen et al., 2009), *ku540* was the only mutation that rescued *elo-5(−)* larvae to adults and permitted growth for more than one generation without mmBCFA supplementation or restoration of mmBCFA synthesis (Figure 2B–E; see ‘Materials and methods’). In fact, *elo-5(−);ku540* homozygous animals could propagate continuously, even though they displayed slow growth, smaller body size, and smaller brood size (Figure 2C).

**Figure 2—figure supplement 1.**
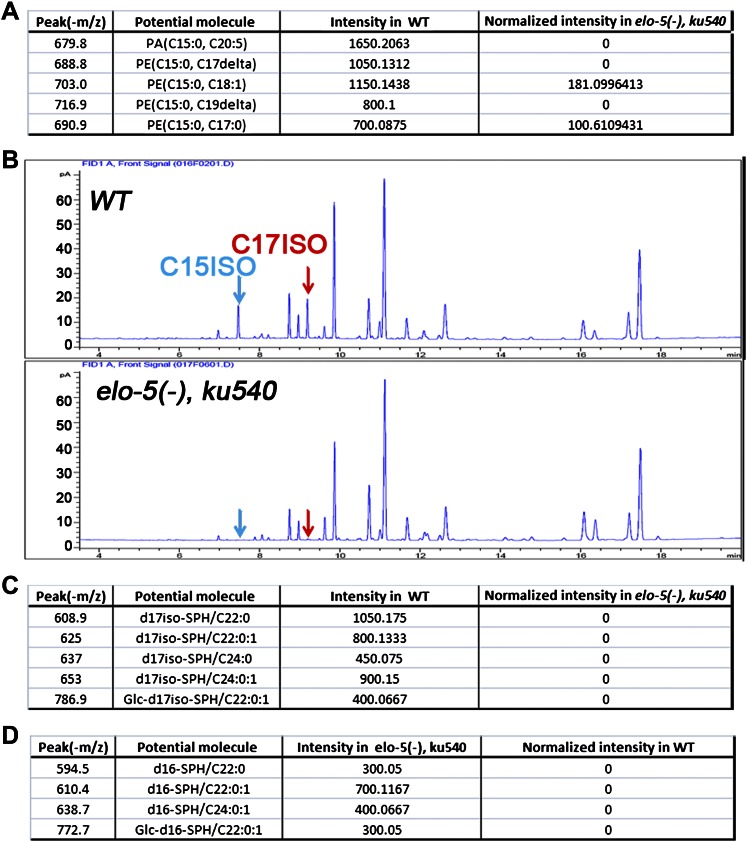
Quantification of GC and mass spectrometry analyses of *elo-5(−) ku540* animals. (**A**) Tables show relative intensities of the five strongest signals by mass spectrometry precursor scan (*m/z* = −241.2) in wild-type animals (second right column) and their normalized relative intensities in *elo-5(−) ku540* animals (right column). The low intensities of these lipids in *elo-5(−) ku540* animals indicate that the C15ISO mmBCFA level was not restored *elo-5(−) ku540* animals. (**B**) Gas chromatography of methyl-esterified fatty acid extracts showing no detectable C15ISO or C17ISO in *elo-5(−) ku540* animals. (**C**) Table shows relative intensities of normal d17iso-sphingolipid by mass spectrometry precursor scan (*m/z* = +250.3) in wild-type animals (second right column) and their normalized relative intensities in *elo-5(−) ku540* animals (right column). The low intensities of these lipids in *elo-5(−) ku540* animals indicate that the d17iso-sphingolipid level was not restored *elo-5(−) ku540* animals. (**D**) Table shows relative intensities of abnormal d16-sphingolipid by mass spectrometry precursor scan (*m/z* = +238.3) in *elo-5(−) ku540* animals (second right column). These sphingolipids are not present in wild-type animals (right column). **DOI:** http://dx.doi.org/10.7554/eLife.00429.005

**Figure 2—figure supplement 2.**
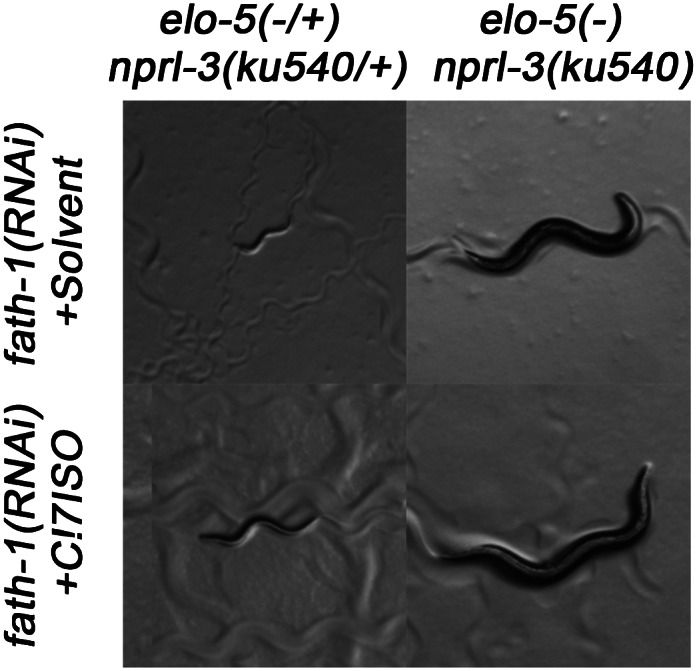
Suppression of *fath-1(−)* by *nprl-3(ku540)*. Microscopic images showing that *nprl-3(ku540)* suppresses the L1 arrest phenotype of *fath-1 (injection RNAi)* with or without C17ISO supplementation. **DOI:** http://dx.doi.org/10.7554/eLife.00429.006

Mass spectrometry (MS) and gas chromatography (GC) analyses indicated that *elo-5(−)*;*ku540* did not restore mmBCFA levels, and consequently the levels of d17iso-sphingolipids (Figure 2D–G). *elo-5(−);ku540* animals had no detectable C15ISO-containing or C17ISO-containing lipids (Figure 2D,E, Figure 2—figure supplement 1A,B). In contrast to wild type animals, *elo-5(-);ku540* animals had ceramides and glucosylceramides containing straight chain d16LCB rather than d17isoLCB (Figure 2F,G, Figure 2—figure supplement 1C,D), which is similar to the *elo-5(−)* single mutant (Entchev et al., 2008). These data indicated that the suppression of L1 arrest by the *ku540* mutation is neither due to a change in mmBCFA nor the metabolism of the derived sphingolipids. Therefore, our data suggest that *ku540* renders mmBCFAs nonessential for *C. elegans* development.

### Mutation *ku540* also permits animals to bypass the requirement for d17iso-GlcCer in postembryonic development

The above results suggested that *ku540* might modify a signaling pathway downstream of d17iso-sphingolipids. If so, *ku540* should also suppress mutations disrupting the biosynthesis of this sphingolipid. This question was addressed by the following tests. As shown above, RNAi of *fath-1,* or mutating both *cgt-*1 and *cgt-3,* caused larval arrest that could not be rescued by feeding with either mmBCFAs or d17iso-SPA (Figure 1A,B). We found that the L1 arrest under both conditions, just like the arrest caused by *elo-5(−)*, was effectively suppressed by *ku540* (Figure 2H,I and Figure 2—figure supplement 2). These results provided strong evidence that d17iso-GlcCer mediated the roles of mmBCFAs and d17iso-SPA in postembryonic development. Taken together, our biochemical and genetic data indicate that *ku540* bypasses the requirement of mmBCFAs and d17iso-GlcCer in development and does so by modifying a regulatory function downstream of d17iso-GlcCer.

### *ku540* is a loss-of-function mutation of *nprl-3* that may function with *nprl-2*

**Figure 3.**
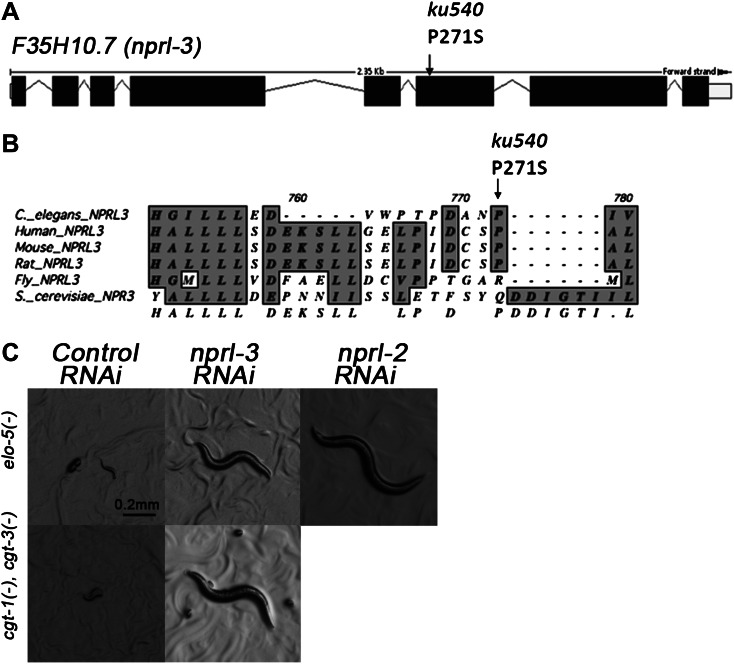
*ku540* is a loss-of-function missense mutation of *nprl-3*. (**A**) Predicted structure and position of the *ku540* mutation in the *nprl-3* gene (F35H10.7). (**B**) Abbreviated alignment of *C. elegans* NPRL-3 with its orthologs in other organisms. (**C**) *C. elegans* images showing *nprl-3(RNAi)* mimics the effect of the *ku540* mutation in rescuing the L1 arrest phenotype caused by blocking mmBCFA or glucosyl-ceramide biosynthesis. *nprl-3* dsRNA injection rescued 61.0% of *elo-5(−)* (n = 123) and 6.8% of *cgt-1(−) cgt-3(−)* (n = 71) animals to or beyond L3 stage. *nprl-2* dsRNA injection also rescued 42.4% of *elo-5(−)* animals to or beyond L3 stage (n = 128). All data have been normalized to heterozygous populations (see ‘Materials and methods’). **DOI:** http://dx.doi.org/10.7554/eLife.00429.007

We cloned the gene containing the *ku540* mutation by genetic mapping and whole-genome sequencing (Figure 3—figure supplement 1A). The gene was annotated as F35H10.7 (wormbase.org) that encodes a protein structurally conserved from budding yeast (NPR3, Nitrogen Permease Regulator 3) to humans (NPRL-3, Nitrogen Permease Regulator Like 3; Figure 3B). We named the gene *nprl-3* (NPRL-3 for protein). *nprl-3(ku540)* is a C-T substitution that changes a conserved proline to serine (Figure 3A). RNAi of *nprl-3* by dsRNA injection also rescued the L1 arrest of *elo-5(−)* and *cgt-1(−)*;*cgt-3(−)* animals (Figure 3C), indicating that *ku540* is likely a partial loss-of-function or reduction-of-function mutation. A transcriptional GFP fusion transgene (*nprl-3::GFP*) was observed to express ubiquitously throughout development and adulthood (Figure 3—figure supplement 1B).

**Figure 3—figure supplement 1.**
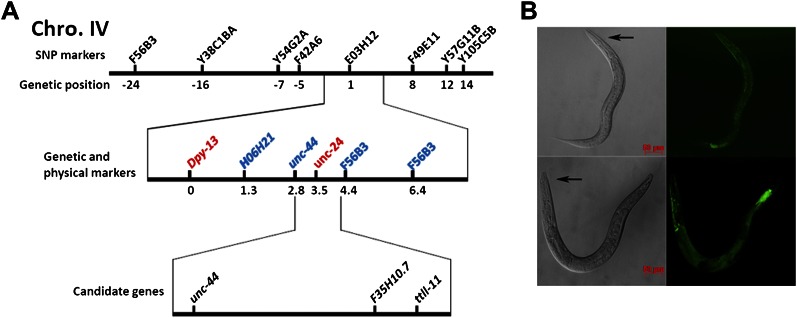
Mapping and expression of *nprl-3*. (**A**) A simplified diagram of the mapping process. *ku540* is genetically linked to the E03H12 SNP marker on chromosome IV (upper section). Further three-point mapping narrowed the *ku540* locus to near *unc-24*, by visible markers (red) and by physical mutations (blue) found by genomic deep sequencing (middle section). The three candidate gene mutations resulting in amino acid changes are shown (lower section). (**B**) DIC and fluorescence images showing two representative animals carrying a *nprl-3* promoter::GFP transgene. The head is indicated by a black arrow. GFP is visible in most tissues with stronger expression seen in the head and tail regions. **DOI:** http://dx.doi.org/10.7554/eLife.00429.008

In *Saccharomyces cerevisiae*, NPR3 was shown to form a heterodimer with NPR2. Mutations in both proteins cause multiple growth defects when cells were cultured in low-quality amino acid conditions (Neklesa and Davis, 2009). We found that RNAi by dsRNA injection of the *C. elegans* NPR2 homolog *nprl-2* (F49E8.1) also suppressed the developmental arrest of *elo-5(−)* larvae (Figure 3C). This suggested that NPRL-2 and NPRL-3 in *C. elegans* may also function in a complex to negatively impact mmBCFA/d17iso-GlcCer-mediated L1 growth.

### NPRL-3 regulates d17iso-GlcCer function in L1 growth by repressing TORC1 activity

The NPR2/3 complex was proposed to be a negative regulator of the TOR pathway in *S. cerevisiae*, based on the observations that mutations in these two genes caused growth defects in an amino acid scarce environment and that the defects could be effectively suppressed by blocking the TOR pathway (Neklesa and Davis, 2009). We thus explored the possibility that TOR Complex 1 (TORC1) is also the downstream target of NPRL2/3 in growth regulation in *C. elegans* and that *nprl-3(ku540)* alleviates d17iso-GlcCer deficiency-induced growth arrest by activating the TORC1 pathway.

We reasoned that if reducing *nprl-2/3* activity rescued *elo*-5*(−)* L1 growth by up-regulating the TORC1 pathway in *C. elegans*, then reducing, but not eliminating, TORC1 activity may reverse the rescue effects of *nprl-3(ku540)* on *elo-5(−)* mutant background. *Caenorhabditis elegans* has orthologs of key components of mammalian TORC1, including regulatory elements *raga*-*1* (RagA) and *rheb*-*1* (Rheb) (Long et al., 2002; Schreiber et al., 2010). Feeding RNAi of *raga-1* and *rheb-1* have been shown to be specific and effective to reduce, but not eliminate, TORC1 function (Hansen et al., 2007; Syntichaki et al., 2007; Lemire et al., 2009; Figure 4—figure supplement 1; see ‘Materials and methods’). We thus tested the effect of *raga-1(RNAi)* and *rheb-1(RNAi)* on *elo-5(−) nprl-3(ku540)* mutants*.*

While *nprl-3(ku540)* effectively suppressed the L1 arrest phenotype of *elo-5(−)*, the suppression dramatically decreased when *raga-1(RNAi)* or *rheb-1(RNAi)* were applied to the *elo-5(−) nprl-3(ku540)* double mutant (Table 1). This indicates that the rescue effect of *nprl-3(ku540)* on *elo-5(−)* depends on intact TORC1 activity.

**Table 1.** Intact TORC1 function is necessary for mmBCFA-mediated growth regulation **DOI:** http://dx.doi.org/10.7554/eLife.00429.009

Genotype	RNAi	Dietary C17ISO	Normalized % of F1 reached adulthood	N	p
*elo-5(−)*	Vector	−	0	208	
*elo-5(−)*	Vector	+	86.9	205	0
*elo-5(−) nprl-3(−)*	Vector	−	71.5	69	
*elo-5(−) nprl-3(−)*	Vector	+	111.5	103	0.24
*elo-5(−) nprl-3(−)*	*raga-1 (a)*	−	35.5	56	
*elo-5(−) nprl-3(−)*	*raga-1 (a)*	+	102.5	83	0.031
*elo-5(−) nprl-3(−)*	*raga-1 (b)*	−	25.5	214	
*elo-5(−) nprl-3(−)*	*raga-1 (b)*	+	88.0	279	0.00001
*elo-5(−) nprl-3(−)*	*rheb-1 (a)*	−	14.2	351	
*elo-5(−) nprl-3(−)*	*rheb-1 (a)*	+	64.6	526	0.0000001
*elo-5(−) nprl-3(−)*	*rheb-1 (b)*	−	32.5	201	
*elo-5(−) nprl-3(−)*	*rheb-1 (b)*	+	86.0	116	0.0037
*elo-5(−) nprl-3(−)*	*rsks-1 (a)*	−	15.0	68	
*elo-5(−) nprl-3(−)*	*rsks-1 (a)*	+	72.5	83	0.022
*elo-5(−) nprl-3(−)*	*rsks-1 (b)*	−	19.0	183	
*elo-5(−) nprl-3(−)*	*rsks-1 (b)*	+	61.0	180	0.0034
*elo-5(−) nprl-3(−)*	*ife-2 (a)*	−	10.5	144	
*elo-5(−) nprl-3(−)*	*ife-2 (a)*	+	71.5	49	0.003
*elo-5(−) nprl-3(−)*	*ife-2 (b)*	−	0	103	
*elo-5(−) nprl-3(−)*	*ife-2 (b)*	+	103.5	150	0
*elo-5(−) nprl-3(−)*	*let-363*	−	0	>100	
*elo-5(−) nprl-3(−)*	*let-363*	+	0	>100	NA

Percentages of *elo-5(−);nprl-3(ku540)* homozygotes with the indicated RNAi treatments that reached adulthood, where *(a)* and *(b)* indicate two different RNAi constructs targeting different parts of the same gene. The presented percentage of *elo-5(−) nprl-3(ku540)* animals that reached adulthood was calculated by normalizing against the percentage of *elo-5(−/+) nprl-3(ku540)*/+ heterozygotes (see ‘Materials and methods’ for detail). Without C17ISO supplementation, RNAi knockdown of multiple TORC1 components reverted *elo-5(−);nprl-3(ku540)* animals to larval arrest.

We carried out further analyses by repeating the above tests in the presence of dietary C17ISO that rescues the defects of *elo-5(−)*. We found that the effects of *raga-1(RNAi)* and *rheb-1(RNAi)* in the above tests were essentially eliminated by C17ISO supplementation (Table 1). This result further indicates that the reversal of the *nprl-3(ku540)* suppression by *raga-1(RNAi)* or *rheb-1(RNAi)* depends on mmBCFA deficiency. In other words, reducing, but not eliminating, TORC1 activity neutralized the effect of *nprl-3(ku540)* and restored the L1 arrest phenotype of *elo-5(−)*.

To further examine whether the rescuing effect of *nprl-3(ku540)* on *elo-5(−)* L1 arrest required the canonical activity of TORC1, we used RNAi to knock down *C. elegans* orthologs of *elf4E* (*ife-2*) and *p70S6K(rsks-1)*, two well-known downstream targets of TORC1 (Long et al., 2002; Syntichaki et al., 2007; Anjum and Blenis, 2008; Sheaffer et al., 2008). We found that *elo-5(−) nprl-3(ku540)* animals treated with *ife-2(RNAi)* or *rsks-1(RNAi),* with or without C17ISO supplement, yielded results similar to those obtained with *raga-1(RNAi)* and *rheb-1(RNAi)* (Table 1). Taken together, these results indicate that TORC1 acts downstream of d17iso-GlcCer and is negatively regulated by NPRL-3*.*

The *let-363/TOR(−)* mutations cause late larval arrest and other severe defects (Long et al., 2002), but injection RNAi causes an early embryonic lethal phenotype (Sonnichsen et al., 2005), indicating that LET-363/TOR, a key component of both TORC1 and TORC2, plays critical regulatory roles in a broad range of developmental stages and a maternal effect largely masks its roles in earlier stages, including its likely functions during L1 growth. The defects caused by feeding RNAi of *let-363/TOR* (Long et al., 2002) are more severe than that by feeding RNAi of other TORC1 components, described above. *elo-5(−) nprl-3(−)*;*let-363(RNAi)* animals displayed pleiotropic phenotypes that could not be rescued by C17ISO supplement (Table 1), which is consistent with TORC1 acting downstream of ELO-5.

### Hyperactivation of TORC1 bypasses mmBCFA deficiency–induced L1 arrest

**Figure 4.**
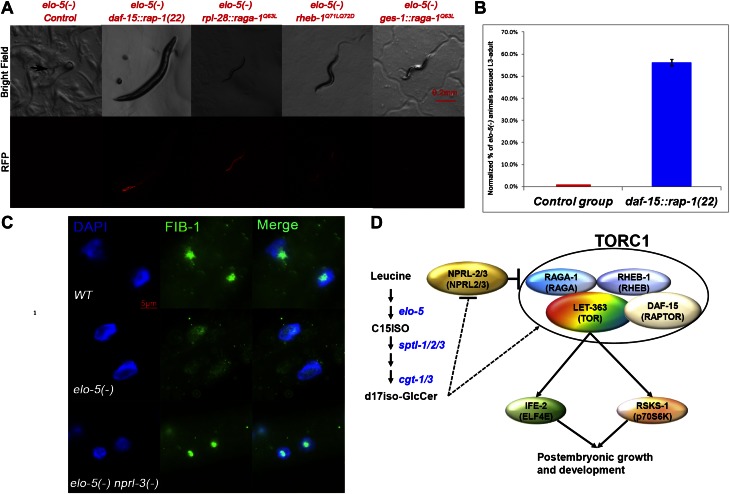
TORC1 activation is sufficient for mmBCFAs-mediated growth regulation. (**A**) Representative florescent images showing that *elo-5(−)* animals with each of four RFP-marked transgenes, which constitutively activated TORC1, bypass L1 arrest to reach beyond L3 stage (statistical data are described in the text and Figure 4B). The *rpl-28* promoter drives ubiquitous expression, whereas the *ges-1* promoter drives the expression specifically in the intestine (Edgar and McGhee, 1986). Arrow in the upper left panel marks an arrested L1. (**B**) Percentage of homozygous *elo-5(−)* animals carrying the *daf-15::rap-1(22)* transgene reached L3-adult stages (n = 161). The data were normalized against that of *elo-5(−/+)* heterozygous animals. Error bar, SD. (**C**). Immunofluorescence images showing FIB-1 expression and localization in intestinal cells of L3 larvae. DAPI-stained nuclei are blue. Green fluorescence indicates the staining of antibody against FIB-1. FIB-1 localization in the nucleoli is largely abolished in *elo-5(−)* animals and restored by the *nprl-3(−)* mutation (the percentages of condensed nucleoli localization for the three genotypes from top to bottom are 95% [n=42], 24% [n=112] and 93% [n=68]). (**D**) A model for the regulation of postembryonic growth and development by mmBCFAs and GlcCer. **DOI:** http://dx.doi.org/10.7554/eLife.00429.010

If d17iso-GlcCer mainly acts through TORC1 for the growth regulation function, constitutive activation of TORC1 should be sufficient to overcome the L1 arrest of *elo*-5*(−)* animals, mimicking the effect of *nprl-3(ku540)*. We used three different established methods to test this possibility. We first followed the scheme by Sancak et al. (2010), in which fusion of one of the TOR binding partners, Raptor, with a C-terminal lysosome localization signal from Rap-1 can localize mTOR to the lysosome and thus constitutively activate the TOR pathway. Both Rap-1 and Raptor proteins are highly conserved in *C. elegans* (encoded by *rap-1* and *daf-15*; Long et al., 2002; Pellis-van Berkel et al., 2005). We fused a 22-amino acid fragment of the C-terminal end of *rap-1* to the C-terminal end of *daf-15* and named it *daf-15::rap-1(22)*. We found that 56.0% of *elo-5(−)* mutants carrying *daf-15::rap-1(22)* bypassed L1 arrest and reached L3 to adult stage (n = 161; normalized against *elo-5(−/+)* heterozygotes containing the transgene; Figure 4A,B). This indicates that hyperactivation of TORC1 was sufficient to support L1 growth in mmBCFA-depleted animals.

**Figure 4—figure supplement 1.**
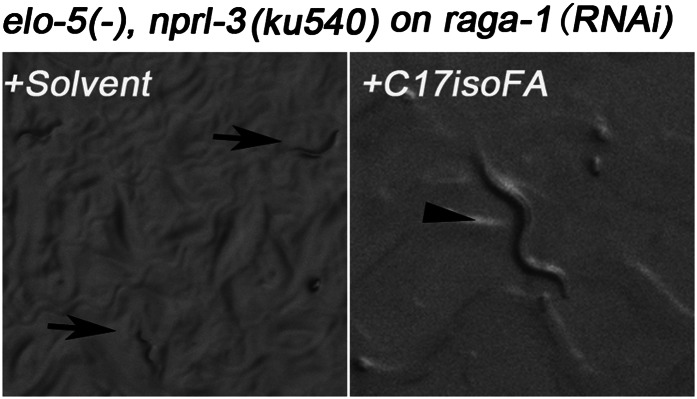
Microscopic images of *C. elegans* with *raga-1(RNAi)* treatment. Animals without C17ISO supplement were developmentally arrested at L1 stage (arrows). Animals with C17ISO supplement reached adulthood (arrowhead), suggesting that the arrest depends on mmBCFA deficiency and is not caused by RNAi itself. **DOI:** http://dx.doi.org/10.7554/eLife.00429.011

**Figure 4—figure supplement 2.**
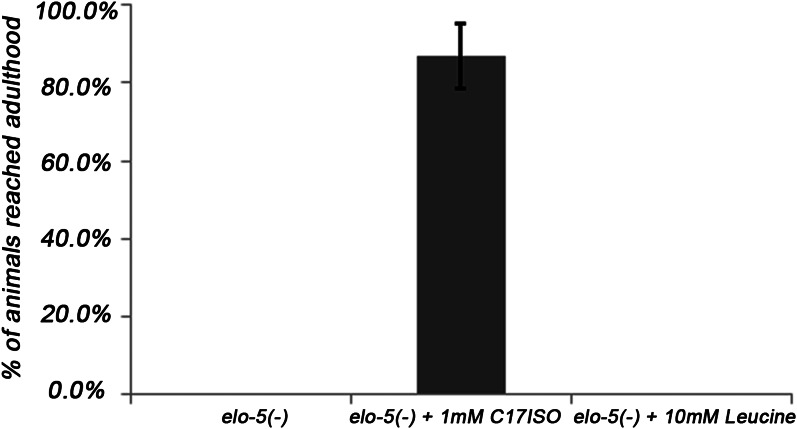
Leucine could not promote postembryonic development independent of the mmBCFA/d17isoGlcCer/TORC1 pathway. Normalized percentages of *elo-5(−)* animals that reached adulthood on various supplements. While 1 mM C17ISO could suppress the L1 arrest of *elo-5(−)*, 10 mM leucine could not. Error bar: SD. **DOI:** http://dx.doi.org/10.7554/eLife.00429.012

**Figure 4—figure supplement 3.**
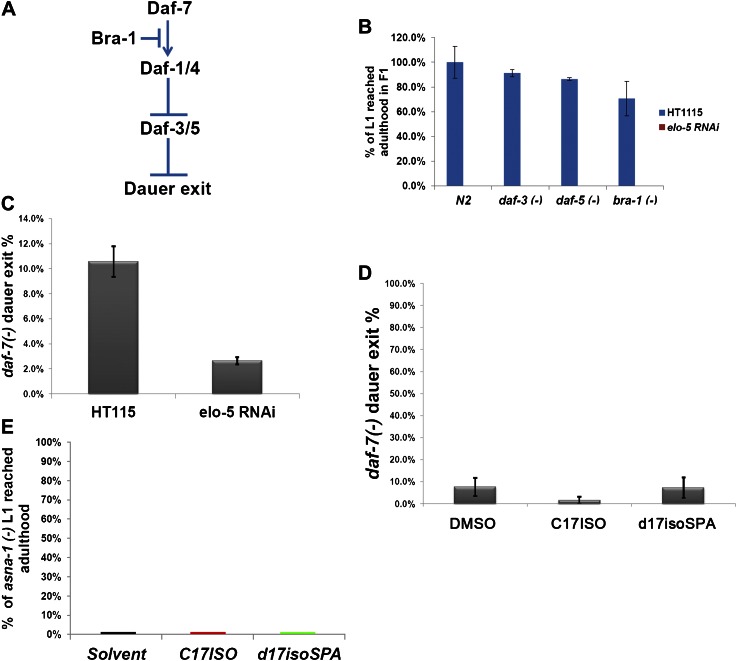
mmBCFA/GlcCer/TORC1 pathway is independent of the DAF-7/TGF-β pathway. (**A**) Cartoon illustration of a simplified DAF-7/TGF-β pathway in *C. elegans*. Mutations in *daf-3*, *daf-5*, or *bra-1* have been shown to cause constitutive activity of the TGF-β pathway and suppress dauer formation (Patterson and Padgett, 2000). (**B**) Percentages of animals of the indicated genotypes that reached adulthood on *elo-5 (RNAi)* plates. Mutation in none of these negative regulators of the *daf-7* pathway permitted the *elo-5(RNAi)*–treated animals to bypass L1 arrest, suggesting that the TGF-β pathway does not act downstream of mmBCFAs. (**C**) Percentages of *daf-7(−)* animals that exited dauer stage to reach adulthood on indicated RNAi plates. *elo-5(RNAi)* enhanced the constitutive dauer formation phenotype of a *daf-7(−)* mutant. (**D**) Percentages of *daf-7(−)* animals that exited dauer stage to reach adulthood on various branched lipid supplements. Neither C17ISO nor d17iso-SPA could suppress the constitutive dauer formation of *daf-7(−)*, suggesting that DAF-7/TGF-β does not act upstream of mmBCFAs or d17iso-sphingolipid. (**E**) Percentages of *asna-1(−)* animals that reached adulthood on plates with various branched-chain lipid supplements. *asna-1* encodes a protein required for proper DAF-7/TGF-β function and an *asna-1(−)* mutation causes L1 arrest (Kao et al., 2007). Neither C17ISO nor d17iso-SPA permits *asna-1(−)* animals to bypass L1 arrest. **DOI:** http://dx.doi.org/10.7554/eLife.00429.013

**Figure 4—figure supplement 4.**
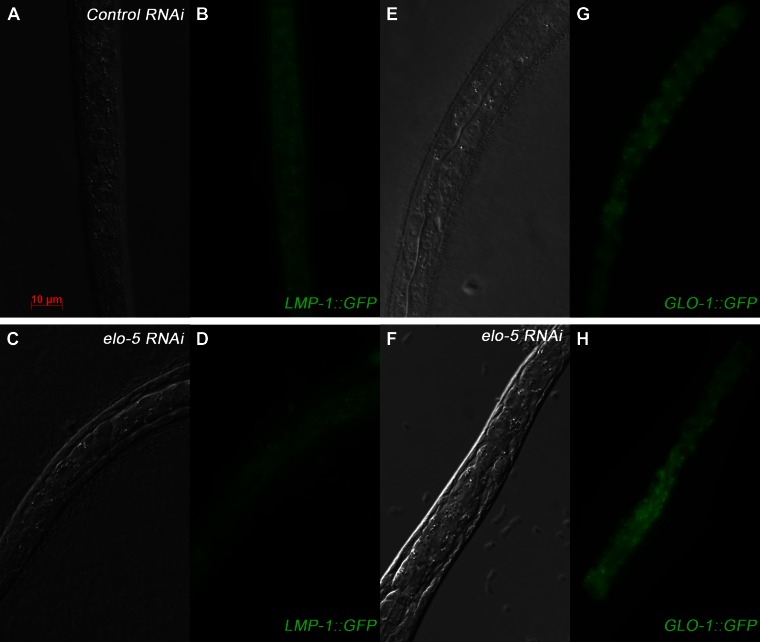
Lysosome integrity is not disrupted in mmBCFA-deficient animals. (**A**)–(**H**) DIC and GFP images illustrating *LMP-1::GFP* (**A**–**D**) and *GLO-1::GFP* (**E**–**H**) expression patterns are similar throughout the intestine of young wild-type or *elo-5*(RNAi) larvae. Both *LMP-1::GFP* (**A**–**D**) and *GLO-1::GFP* are lysosomal markers in *C. elegans*. These data indicate that lysosomal integrity is not disrupted in mmBCFA-deficient animals. **DOI:** http://dx.doi.org/10.7554/eLife.00429.014

**Figure 4—figure supplement 5.**
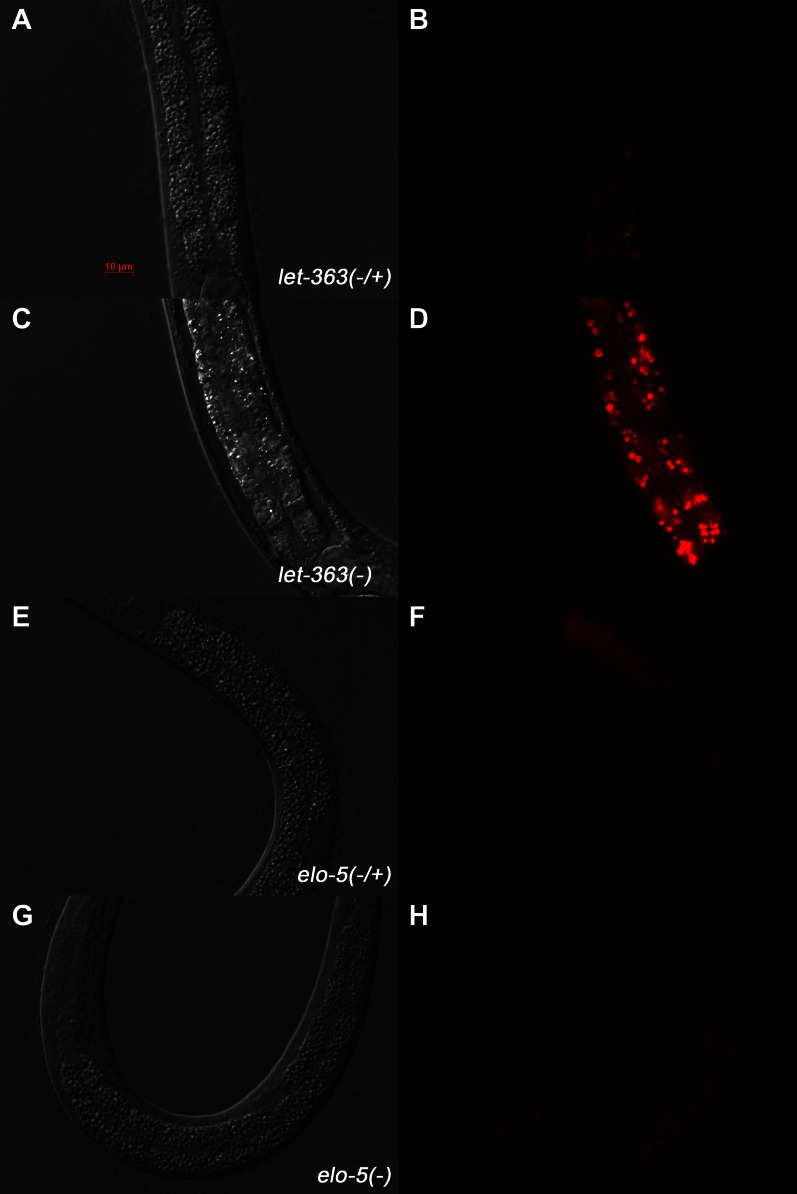
Neutral red staining of *let-363* -and *elo-5*-deficient animals. (**A**)–(**H**) DIC and Rhodamine channel fluorescence images of larvae stained with Neutral red. (**A**–**D**) *let-363* homozygous L3 animals have increased size and intensity of Neutral red stained lysosomes (**D**) compared to the heterozygous control (**B**). (**E**–**H**) *elo-5* homozygous L1 arrested animals show similar Neutral red intensity and staining pattern (**H**) when compared to the heterozygous control (**F**). These data indicate that (1) lysosome integrity is not disrupted in *elo-5(−)* animals, and (2) unlike *let-363(−)*, *elo-5(−)* animals do not show increased Neutral red staining in the intestine. **DOI:** http://dx.doi.org/10.7554/eLife.00429.015

The second TORC1-activating transgene used was *raga-1*^*Q63L*^ that has been proven to constitutively activate TORC1 both in mammals and *C. elegans* (Kim et al., 2008; Schreiber et al., 2010). We found that 50.4% of *elo-5(−)* mutants containing the *raga-1*^*Q63L*^ transgene bypassed L1 arrest and reached L3–L4 stage (n = 248, p=1.0 × 10^−6^; normalized against *elo-5(−/+)* heterozygotes containing the transgene; Figure 4A) while none of *elo-5(−)* mutants without this transgene did (n = 136). Finally, we also tested a third TORC1-activating transgene, *rheb-1*^*Q71LQ72D*^, and found that it also permitted 41.4% of *elo-5(−)* animals to grow beyond the L1 stage (n = 169; normalized against *elo-5(−/+)* heterozygotes containing the transgene; Figure 4A). These results led to the conclusion that TORC1 acts downstream of d17iso-GlcCer to promote L1 growth and development and that this activity is negatively regulated by NPRL-2/3.

### The mmBCFA/d17iso-GlcCer/TORC1 pathway acts in the intestine to promote postembryonic development

Previous studies on *elo-5*, *acs-1, tat-2,* and *cgt-1/cgt-3* mutants suggested that biosynthesis and localization of mmBCFAs and their derived sphingolipids in the intestine are essential and sufficient for postembryonic development (Marza et al., 2009; Seamen et al., 2009; Kniazeva et al., 2012). The fact that *elo-5(−);nprl-3(ku540)* animals treated with *ife-2* RNAi, that targets intestinal but not neuronal expression of the gene (Syntichaki et al., 2007), are L1 arrested (Table 1) also points to the requirement of TORC1 activity in the intestine. To directly evaluate a role of intestinal TORC1 in mmBCFA/d17iso-GlcCer regulated growth, we made a *raga-1*^*Q63L*^ TORC1 hyperactivating transgene driven by the intestinal-specific promoter *ges-1* (Edgar and McGhee, 1986). We found that *elo-5(−)* animals containing this transgene bypassed the L1 arrest (37.9%, n = 132, normalized against *elo-5(−/+)* heterozygotes containing the transgene; Figure 4A). The rescue with the intestinal expression was similar to that by a ubiquitously expressed *raga-1*^*Q63L*^ transgene (above). A neuronal-specific *rgef-1* promoter–driven (Brignull et al., 2006) *raga-1*^*Q63L*^, however, could not suppress the L1 arrest of *elo-5(−)* animals (0%, n > 100). These results suggest that the intestinal d17iso-GlcCer/TORC1 pathway regulates postembryonic growth.

Although the genetic data described above sufficiently indicate that TORC1 acts downstream of d17iso-GlcCer and NPRL-2/3 for L1 growth, we carried out further tests to observe downstream activity of TORC1 in the intestine. Because the biochemical assay for p70S6K phosphorylation has not been established in *C. elegans*’ TOR-related studies, we adopted the strategy by Sheaffer et al. (2008), where localization of FIB-1, a Box C/D small nucleolar ribonucleoprotein (snoRNP) was used as a marker of TORC1 activation in *C. elegans*. In wild-type animals, FIB-1 is highly expressed and localized in the nucleolus, where it methylates pre-rRNAs during ribosome maturation, but the expression is decreased and no longer localized to the nucleolus in *let-363/TOR(−)* animals (Sheaffer et al., 2008). We found that the nucleolar localized FIB-1 was dramatically reduced in intestinal cells of mmBCFA-deficient [*elo-5(RNAi)*] animals (Figure 4C). Furthermore, *nprl-3(−)* rescued the FIB-1 expression and nucleolar localization in *elo-5(−)* back to that of wild type (Figure 4C). Combining the results from the functional and biochemical assays described above, we conclude that TORC1 is the key downstream factor mediating mmBCFA and d17iso-GlcCer functions in the intestine to promote postembryonic growth and development (Figure 4D).

## Discussion

### Roles of the mmBCFA/GlcCer/TORC1 pathway in promoting postembryonic development

In this study, we uncovered and characterized a novel GlcCer-stimulated TORC1 pathway that promotes postembryonic development (Figure 4D). This discovery reveals a specific link between lipids and the TORC1 signaling pathway. Both ceramide and TORC1 have been individually implicated for roles in stress response, apoptosis, cancers, and other cellular processes by studies in *C. elegans* and mammals (Hannun and Obeid, 2008; Hansen et al., 2008; He and Klionsky, 2009; Howell and Manning, 2011; Laplante and Sabatini, 2012; Menuz et al., 2009; Zoncu et al., 2011). It is conceivable that some of these functions are mediated by this GlcCer/TORC1 signaling pathway. Moreover, our data suggest that this GlcCer/TORC1 pathway may serve as a ‘check point’ to coordinate metabolic status in the intestine with postembryonic growth and development. d17iso-GlcCer is a sphingolipid metabolite rather than a nutrient directly absorbed from food (Merrill et al., 1997); it is the product of a long biosynthetic pathway involving many enzymatic steps and thus may reflect the availability of many metabolites and enzymes.

Although leucine is a precursor molecule of mmBCFA biosynthesis, this long lipid synthesis pathway is unlikely part of a mechanism to specifically sense the level of essential amino acids. Furthermore, mmBCFA levels are stable in starved L1 larvae (Kniazeva et al., 2008; Figure 4—figure supplement 4C), suggesting that mmBCFA levels do not directly reflect the feeding status through a direct substrate–product relationship. There are several reported leucine-sensing mechanisms, based on studies using tissue culture cells (Bonfils et al., 2012; Duran et al., 2012; Han et al., 2012; Zoncu et al., 2012). To test if such a pathway acts in parallel to the mmBCFA/d17iso-GlcCer pathway to promote TORC1 in the *C. elegans* intestine, we supplemented *elo-5(−)* animals with a high level of dietary leucine (10 mM) and failed to observe any suppression of the L1 arrest phenotype (Figure 4—figure supplement 2), consistent with the idea that TORC1 activation by mmBCFA/d17iso-GlcCer is essential in the intestine of L1 larvae and cannot be effectively compensated by another leucine/TORC1 mechanism in *C. elegans*.

In *C. elegans*, both the IIS and DAF-7/TGF-β pathways have been shown to play critical roles in the regulation of postembryonic growth and development in response to nutrient/food availability (Baugh and Sternberg, 2006; Mukhopadhyay and Tissenbaum, 2007; Fielenbach and Antebi, 2008; Lee and Ashrafi, 2008; Jones et al., 2009; Soukas et al., 2009). Our previous data and this study indicated that the GlcCer/TORC1 pathway is initiated in the intestine (Figure 4) and is independent of the IIS pathway (Kniazeva et al., 2008). Our additional analysis also suggests that the GlcCer/TORC1 pathway is DAF-7/TGF-β pathway independent (Figure 4—figure supplement 3). This independence may facilitate the role of such a pathway to promote specific developmental events under specific physiological conditions.

### The GlcCer/TORC1 signaling pathway is likely conserved in mammals

There are several reasons for us to believe that such a GlcCer/TORC1 pathway is likely conserved in mammals. First, the components of TORC1 are conserved between *C. elegans* and mammals. The NPRL-3 protein we identified in this pathway is conserved in all eukaryotes, and its negative regulatory role on TOR has been characterized in budding yeast (Neklesa and Davis, 2009). Several proteins (such as NPR2 and IML1) that are reported to form a complex with NPR3 in budding yeast have orthologs in *C. elegans* as well as mammals (Neklesa and Davis, 2009; Wu and Tu, 2011). NPRL2 (human ortholog of NPR2) and DEPDC5 (human ortholog of IML1) have been reported to function as tumor suppressors (Merrill et al., 1997; Li et al., 2004; Neklesa and Davis, 2009). This property is consistent with the role of this complex in repressing TOR-mediated growth regulation that was identified in budding yeast and this study. Second, a role for GlcCer in nutrient sensing is consistent with studies showing that ceramide and sphingolipids play roles in cell signaling and growth control in both invertebrates and mammals (Deng et al., 2008; Hannun and Obeid, 2008; Menuz et al., 2009). The finding that mouse mutants with blocked GlcCer biosynthesis die as early embryos suggests the essential role of these lipids in growth and development (Yamashita et al., 1999). Third, the TOR pathway is down-regulated by inhibiting the abnormally high GlcCer biosynthesis in polycystic kidney disease in mouse models (Natoli et al., 2010), suggesting a possible conserved link between GlcCer and TOR activities.

It is currently unknown whether an iso-branched LCB is also required for the potential role of GlcCer in mammals, given that most LCBs in mammals are straight-chain LCBs. However, it is important to note that mmBCFAs (derived from branched-chain amino acids) are also present in mammals. For example, they are constituents of sphingolipids in skin cells and the intestinal tract of human newborns. They are also found to be incorporated into LCBs (to form an iso- or an anteiso-LCB similar to that in *C. elegans*) in multiple mammalian tissues (Aungst, 1989; Karlsson, 1997; Oku et al., 2000; Ran-Ressler et al., 2008). Therefore, their important physiological functions, including possible roles in ceramide-involved growth regulation, may be assumed, albeit not yet uncovered experimentally.

### Activation of TORC1 by d17iso-GlcCer

How does d17iso-GlcCer regulate the activity of TORC1? Recent work in mammalian cells has indicated that TORC1, for its role in sensing amino acids, localizes at the surface of the lysosome, likely in lipid rafts (Nada et al., 2009). If such a mechanism is conserved in all animals, conceptually, d17iso-GlcCer could act as a ligand that binds to a ‘receptor’ to either repress the NPRL-2/3 complex or activate the TOR complex at the surface of the lysosome. Alternatively, this lipid could be required for lysosome biogenesis or could be an essential constituent of the membrane microdomain that permits the proper localization or activity of TORC1 on the lysosome. By examining lysosomal markers LMP-1::GFP (Artal-Sanz et al., 2006; O’Rourke et al., 2009; Rabbitts et al., 2008), GLO-1::GFP (Schroeder et al., 2007; Zhang et al., 2010), and Neutral red (Long et al., 2002), we did not observe obvious defects in lysosome formation in *elo-5(−)* mutants (Figure 4—figure supplements 4 and 5). Major defects in lysosome formation would also be inconsistent with our genetic suppression data and previous studies on GlcCer by others (Entchev et al., 2008; van der Poel et al., 2011). One possible mechanism is that d17iso-GlcCer acts through the V-ATPase pathway, since the lysosomal V-ATPase has been shown to be stimulated by GlcCer and to play an essential role in TORC1 activation in studies using tissue culture cells (van der Poel et al., 2011; Zoncu et al., 2011; Bar-Peled et al., 2012). Further biochemical and genetic analyses are needed to test this hypothesis in *C. elegans*.

It may be important to point out again that activation of TORC1 bypasses the robust L1 arrest phenotype caused by either mmBCFA or d17iso-GlcCer deficiency. Remarkably, the *elo-5(−); nprl-3(ku540)* double mutants, still deficient for these lipids, propagate continuously. Therefore, the only essential biochemical role of d17iso-GlcCer might be to activate TORC1 or repress NPRL-3. In other words, the biochemical mechanism underlying this role of d17iso-GlcCer appears to be very specifically connected to TORC1 activation.

## Materials and methods

### *Caenorhabditis elegans* strains and maintenance

The following strains were obtained from the Caenorhabditis Genetics Center Database (CGC) or as indicated; wild-type N2 Bristol, *elo-5(gk208), rrf-3(pk1426), daf-3(mgDf90), bra-1(nk1), daf-5(e1386), asna-1(ok938)/hT2[bli-4(e937)let?(q782)qIs48], let-363 (ok3018), pwIs50[lmp-1::GFP + Cbr-unc-119(+)], hjIs9 [ges-1p::glo-1::GFP + unc-119(+)].* The *cgt-1(tm1027)* and *cgt-3(tm504)* mutants were provided by the Mitani Lab (National BioResource Project, Tokyo, Japan). *Caenorhabditis elegans* were maintained at 20°C on NGM plates (referred to as standard plates) with *Escherichia coli* OP50 bacterial food (OP50/NGM). Washes and bleaching were done according to standard protocols (Stiernagle, 2006).

### Dietary supplements

Fatty acids C13ISO, C17ISO (Larodan), C16:1 n7, C18:1 n7, and C20:4 n6 (Sigma), as well as C17iso-d18:1-Ceramide and d17iso-SPA (custom synthesis; Larodan) were prepared as 10 mM stocks in DMSO. Leucine (Sigma) was prepared as a 100 mM stock in water. A stock solution was mixed with 500 µl of OP50 overnight bacterial suspension in a 1:10 ratio.

### Preparation of the C17ISO-deficient eggs and larvae

- Using *elo-5(gk208)* mutants. *elo-5(gk208)* adults maintained on the plates with *S. maltophlia* (Kniazeva et al., 2008) were washed off in M9 buffer, bleached, and eggs were plated on NGM plates spotted with 300 µl of OP50 overnight liquid culture supplemented with 1 mM C13ISO, which is a less efficient mmBCFA supplement than C17ISO and allows apparent normal growth only in the first generation followed by uniformly arrested C17ISO-deficient L1s in the second generation. Control animals were prepared in the same way, except C17ISO was used as a supplement for the N2 strain instead of *elo-5(gk208).*
- Using RNAi. Adult animals of the corresponding strains were washed off OP50/NGM plates, bleached, and eggs plated on *elo-5(RNAi)* plates prepared according to the standard protocol. Adults of the next generation were bleached for eggs, producing C17ISO-deficient larvae.

### Preparation of C17ISO-deficient late larvae and adults

A mixed population of *elo-5(gk208)* mutants maintained on plates with C17ISO supplement, which promoted wild-type growth and proliferation, were collected, washed, and incubated for 1 hr in M9 before replating on OP50/NGM plates without supplement. The next day, animals were washed off and used in the corresponding experiments. Depletion of C17ISO was confirmed by GC analysis of the FA composition in total lipid extracts from a representative group of animals.

### Lipid analysis by gas chromatography and mass spectrometry

Gas chromatography method was described in a previous report (Kniazeva et al., 2008). Mass spectrometry sample preparation was described in our previous report (Kniazeva et al., 2012). Briefly, lipid extracts were dissolved in 1 ml of methanol with 1 mM formic acid and subjected to quantitative lipid analysis using a 4000 Q-Trap mass spectrometer (AB Sciex). Samples were infused at a flow rate of 8 μl/min using a Harvard Apparatus syringe pump (Harvard Apparatus). The detailed scan modes are described in the figure legends.

### Purification of d17iso-LCB from *Sphingobacterium spiritivorum*

The *S. spiritivorum* strain was obtained from ATCC (#33861) and cultured under the conditions suggested by www.atcc.org. The total lipids were extracted by the method of Bligh and Dyer (1959). The alkaline stable lipid fraction was purified as described previously (Naka et al., 2003). The hydrolysis of sphingolipids to generate side chain fatty acid and LCB fractions was done as described previously (Naka et al., 2003), with an additional last step using 100% methanol to extract the dry LCB fraction after evaporation of the chloroform solvent. The final fraction of LCB was verified by mass spectrum.

### Isolation of *nprl-3(ku540)* through a genetic screen

We performed a screen that was significantly different from our previously published screen that resulted in isolation of three *tat-2(−)* alleles that suppress *elo-5(gk208)* (Seamen et al., 2009). The original strain used in this genetic screen was *elo-5(gk208)* injected with *elo-5(genomic rescuing), elo-5Promoter::GFP* (Kniazeva et al., 2004), and *rol-6(dn)* constructs. L4-staged P_0_ animals were mutagenized with standard EMS treatment and then single cloned to 10-cm plates containing 1 mM C13ISO. Suppressor candidates were determined in the F2 generation by the ability to reach gravid adulthood without carrying the GFP or *rol-6* marker and the ability to grow to gravid adulthood without any supplement. From ∼2000 haploid genomes, 4 suppressor candidates were isolated. Among these four, and several suppressor mutations isolated in a previous screen (Seamen et al., 2009), only *ku540* permits *elo-5(−)* animals to grow indefinitely without any mmBCFA supplementation and without recovering the production of mmBCFAs.

In our previously published screen (Seamen et al., 2009), we isolated three mutations in *tat-2* that can suppress *elo-5(−)*–induced L1 arrest in the presence of C13ISO, or temporarily suppress the L1 arrest for one generation without C13ISO supplement. The hypothesis was that *tat-2(−)* alters the subcellular localization of certain mmBCFA-containing lipids so that their levels (from a slow C13ISO to C17ISO conversion or from maternal sources) are sufficiently maintained for one more generation. Therefore, *tat-2(−)* does not bypass the requirement of mmBCFAs for growth. The *tat-2(−)* mutations were also found to partially and temporarily suppress the lethality associated with disrupting the function of the enzyme (SPTL-1) in the first step of sphingolipid biosynthesis, suggesting a potential link between sphingolipids and mmBCFAs. However, this observation may not be interpreted as the proof that mmBCFAs function through sphingolipids for the following reasons. (1) Unlike feeding d17iso-SPA or the downstream suppressor *nprl-3(ku540)* that fully suppresses mmBCFA deficiency [*elo-5(−)*]–induced L1 arrest (this study), *tat-2(−)*does not bypass the requirement of mmBCFAs for growth for more than one generation. (2) *sptl-1(−)* disrupts all sphingolipid biosynthesis and its phenotype is, in contrast to *elo-5(−)*, highly pleiotropic, as animals showed various morphological and growth defects and die at various larval stages. (3) *tat-2* does not exclusively function with mmBCFA-containing lipids; it is also a critical player in steroid metabolism as exemplified by a recent article (Liu et al., 2012). Therefore, the link between mmBCFA and sphingolipids through their interactions with *tat-2* could be indirect and the partial suppression of *sptl-1(−)* by *tat-2(−)* could be due to suppression of mmBCFA-unrelated functions of sphingolipids.

### SNP mapping of *ku540* and cloning of the *nprl-3* gene

For SNP mapping of *ku540*, we used a Hawaii strain based *elo-5(gk208)* mutant (Seamen et al., 2009) to cross with *elo-5(gk208) ku540* animals and determined the locus of *ku540* by linkage analysis (Davis et al., 2005). The three-point-mapping strain *dpy-13(e184)elo-5(gk208)unc-24(e138)* was also used in this study. The *elo-5(−)ku540* genomic DNA was prepared using the Genomic DNA Sample Preparation Kit (Illumina) and sent for Illumina deep sequencing (High Throughput Next-Generation Sequencing Core, University of Colorado). The raw data was analyzed using Maqgene (Bigelow et al., 2009), and candidate mutations were confirmed by PCR and sequencing. Homologs of NPRL-2 and NPRL-3 were identified at ensembl.org. The alignment among NPRL3 homologs was done using MacVector software.

### Analysis of *elo-5(gk208) nprl-3(ku540)* double mutants

Except for Figure 2I, all *elo-5(−) nprl-3(ku540)* animals being tested for Figure 2 were from homozygous mothers. For Figure 2I, because the control *cgt-3(−);cgt-1(−)* animals were fully arrested at L1 and were derived from *cgt-3(−);cgt-1(−)/nT1[qIs51]* heterozygotes, we had to use heterozygous *cgt-3(−);elo-5(−) nprl-3(ku540)/nT1[qIs51];cgt-1(−)/nT1[qIs51]* P_0_ animals to generate *cgt-1(−);cgt-3(−);elo-5(−) nprl-3(ku540)* homozygous animals to generate comparable data. Because it is difficult to determine the genotype of arrested L1 animals, the ratios of homozygous *cgt-1(−);cgt-3(−);elo-5(−) nprl-3(ku540)* animals that reached adulthood were calculated by the ratio of the homozygous animals in the total adult population, with a normalization by dividing by the expected ratio of 20%, from the Mendelian distribution of strains containing a recessive lethal translocation balancer (Edgley et al., 2006).

Generation of *elo-5(−) nprl-3(ku540)* homozygous animals for the tests shown in Table 1 and Figure 4B is described below.

### RNAi analysis by feeding and injection

All RNAi by feeding, except *cgt-3(RNAi)* and *let-363(RNAi)*, used bacterial clones from the MRC RNAi library (Kamath et al., 2003) or the ORF-RNAi Library (Open Biosystems) (Figures 1–3). *cgt-3(RNAi)* and *let-363(RNAi)* constructs were made as described (Marza et al., 2009; Honjoh et al., 2009). Feeding RNAi experiments were done as previously described (Kniazeva et al., 2008). The DNA templates for *nprl-3* and *nprl-2* dsRNA synthesis were amplified from the RNAi-containing bacterial strain (MRC RNAi library) by PCR using T7 primers. dsRNA was synthesized using a MEGAscript RNAi Kit (Life Technologies) and then injected into adults of *elo-5(−)/nT1[qIs51], elo-5(-)nprl-3(ku540)/nT1[qIs51],* or *cgt-3(-);cgt-1(-)/nT1[qIs51]*. The eggs were collected from the 8th to 24th hr after injection. Nongreen F1 adult animals were verified by PCR to confirm the homozygosity of *elo-5(−)* or *cgt-1(−);cgt-3(−)* animals.

### Feeding RNAi analysis of TORC1 components and downstream targets

All RNAi (*raga-1, rheb-1, rsks-1, ife-2*) by feeding used sequence confirmed bacterial clones from both the MRC RNAi library (Kamath et al., 2003) or the ORF-RNAi Library (Open Biosystems) (Table 1). These RNAi clones have been extensively used for TORC1-related studies in many publications/meeting abstracts, and their efficiency and specificity have been well established. Furthermore, results from those references and our experiments have shown RNAi feeding of these genes did not reproduce the strong larval lethal phenotype (Hansen et al., 2007; Syntichaki et al., 2007; Lemire et al., 2009; Ching et al., 2010; Polley and Fay, 2012). This difference indicates that TORC1 function is not completely eliminated by RNAi feeding of these genes (*raga-1, rheb-1, rsks-1, ife-2*).

The *elo-5(−) nprl-3(ku540)* animals were balanced by a GFP-labeled *nT1[qIs51]* balancer and treated with feeding RNAi. In the next generation, similar to the method described above, the ratios of homozygous *elo-5(−) nprl-3(−)* animals that reached adulthood were calculated by the ratio of homozygous *elo-5(−) nprl-3(−)* animals in the total adult population, with a normalization by dividing by the expected ratio 20%. As reported, RNAi knockdown of TORC1 components and the downstream target genes would affect the normal development of *C. elegans* (Long et al., 2002; Syntichaki et al., 2007; Zoncu et al., 2011). In our experiments, the usage of the heterozygous *elo-5(−) nprl-3(ku540)/nT1[qIs51]* animals for the RNAi knockdown experiment allowed us to exclude the potential negative effect from RNAi treatment of TORC1 components and TORC1 target genes themselves. Any of those *elo-5(−) nprl-3(−)–*independent negative effects from those RNAi treatments would affect the heterozygous as well as the homozygous *elo-5(−) nprl-3(−)* animals, and therefore not be included in the ratio in the data presented in Table 1.

### Generation of transgenes

For the *pPD95.77-nprl-3*::GFP plasmid, we cloned the potential 1 kbps promoter region upstream of the operon containing *nrpl-3* and inserted it into the pPD95.77 vector (Figure 4A,B). For *raga-1*^*Q63L*^, the genomic DNA including the full coding region was cloned into pPD95.77, driven by a ubiquitous RPL28 promoter. For *ges-1::raga-1*^*Q63L*^, the genomic DNA including the full coding region was cloned into pPD95.77, driven by an intestinal-specific *ges-1* promoter. For *rheb-1*^*Q71LQ72D*^, the genomic DNA including the full coding region and about 1 kbps of upstream sequence was cloned into pPD95.77. Amino acid mutations in both mutants were introduced by replacement with PCR-generated DNA fragments containing the designed mutations. For *daf-15::rap-1(22)*, the genomic DNA of *daf-15*, including the full coding region and the 1 kbps potential promoter region upstream of *daf-15*, was cloned from the fosmid WRM061cH04. After the stop codon of *daf-15* was removed, it was fused with the genomic DNA encoding the last 22 amino acids and the 3′ UTR of *rap*-*1*. For *pPD95.77-nprl-3*::GFP, 25 ng/μl of plasmid was injected in wild-type animals. For the *daf-15::rap-1(22)* rescue experiment, *elo-5(−)/nT1[qIs51]* animals were injected with 10 ng/µl *daf-15::rap-1(22)* and 25 ng/µl *psur-5*::RFP plasmid. In the next generation, the ratios of homozygous *elo-5(−)* animals that reached L3-young adulthood were calculated by the ratio of homozygous *elo-5(−) nprl-3(−)* animals in RFP-positive L3-young adult population, with a normalization by dividing by the expected ratio 20%, as described above. The reason for using heterozygous *elo-5(−)/nT1[qIs51]* animals for this experiment is similar to that for the RNAi knockdown experiment we described above. By this method, we exclude the negative effect we observed from constitutively active TORC1 transgenes (*daf-15::rap-1(22), raga-1*^*Q63L*^*, ges-1::raga-1*^*Q63L*^ *or rheb-1*^*Q71LQ72D*^) for their rescue effects.

### Neutral red staining

The Neutral red staining was performed following Long et al. (2002). Animals were fed with Neutral red containing bacteria food for <10 min before evaluation by microscopy.

### Microscopy

Analysis of GFP expression and phenotypic abnormalities were performed with Nomarski optics using a Zeiss Axioplan2 microscope and a Zeiss AxioCam MRm CCD camera. Plate phenotypes were observed using a Leica MZ16F dissecting microscope, and pictures were taken with a Hamamatsu C4742-95 CCD camera.

### Statistical analysis

All statistical analyses, except the dsRNA feeding for TOR-related genes, were performed using Student’s *t*-test, and p<0.05 was considered a significant difference. The Fisher’s exact test was used for analysis of the TOR-related dsRNA feeding experiments and *raga-1*^*Q63L*^ rescue experiment, and p<0.05 was considered a significant difference.

## Additional files

Supplementary file 1. Detailed description of phenotypes listed in Figure 1B. **DOI:** http://dx.doi.org/10.7554/eLife.00429.016

## References

[bib1] Anjum R Blenis J 2008 The RSK family of kinases: emerging roles in cellular signalling. Nat Rev Mol Cell Biol 9:747–58 doi: 10.1038/nrm2509 18813292

[bib2] Artal-Sanz M Samara C Syntichaki P Tavernarakis N 2006 Lysosomal biogenesis and function is critical for necrotic cell death in *Caenorhabditis elegans*. J Cell Biol 173:231–9 doi: 10.1083/jcb.200511103 16636145 PMC2063814

[bib3] Aungst BJ 1989 Structure/effect studies of fatty acid isomers as skin penetration enhancers and skin irritants. Pharm Res 6:244–7 doi: 10.1023/A:1015921702258 2726682

[bib4] Bar-Peled L Schweitzer LD Zoncu R Sabatini DM 2012 Ragulator is a GEF for the rag GTPases that signal amino acid levels to mTORC1. Cell 150:1196–208 doi: 10.1016/j.cell.2012.07.032 22980980 PMC3517996

[bib5] Baugh LR Sternberg PW 2006 DAF-16/FOXO regulates transcription of cki-1/Cip/Kip and repression of lin-4 during *C. elegans* L1 arrest. Curr Biol 16:780–5 doi: 10.1016/j.cub.2006.03.021 16631585

[bib6] Bigelow H Doitsidou M Sarin S Hobert O 2009 MAQGene: software to facilitate *C. elegans* mutant genome sequence analysis. Nat Methods 6:549 doi: 10.1038/nmeth.f.260 19620971 PMC2854518

[bib7] Bligh EG Dyer WJ 1959 A rapid method of total lipid extraction and purification. Can J Biochem Physiol 37:911–7 doi: 10.1139/o59-099 13671378

[bib8] Bonfils G Jaquenoud M Bontron S Ostrowicz C Ungermann C De Virgilio C 2012 Leucyl-tRNA synthetase controls TORC1 via the EGO complex. Mol Cell 46:105–10 doi: 10.1016/j.molcel.2012.02.009 22424774

[bib9] Brignull HR Moore FE Tang SJ Morimoto RI 2006 Polyglutamine proteins at the pathogenic threshold display neuron-specific aggregation in a pan-neuronal *Caenorhabditis elegans* model. J Neurosci 26:7597–606 doi: 10.1523/JNEUROSCI.0990-06.2006 16855087 PMC6674286

[bib10] Ching TT Paal AB Mehta A Zhong L Hsu AL 2010 drr-2 encodes an eIF4H that acts downstream of TOR in diet-restriction-induced longevity of *C. elegans*. Aging Cell 9:545–57 doi: 10.1111/j.1474-9726.2010.00580.x 20456299 PMC2910166

[bib11] Chitwood DJ Lusby WR Thompson MJ Kochansky JP Howarth OW 1995 The glycosylceramides of the nematode *Caenorhabditis elegans* contain an unusual, branched-chain sphingoid base. Lipids 30:567–73 doi: 10.1007/BF02537032 7651085

[bib12] Davis MW Hammarlund M Harrach T Hullett P Olsen S Jorgensen EM 2005 Rapid single nucleotide polymorphism mapping in *C. elegans*. BMC Genomics 6:118 doi: 10.1186/1471-2164-6-118 16156901 PMC1242227

[bib13] Deng X Yin X Allan R Lu DD Maurer CW Haimovitz-Friedman A 2008 Ceramide biogenesis is required for radiation-induced apoptosis in the germ line of *C. elegans*. Science 322:110–5 doi: 10.1126/science.1158111 18832646 PMC2585063

[bib14] Duran RV Oppliger W Robitaille AM Heiserich L Skendaj R Gottlieb E 2012 Glutaminolysis activates Rag-mTORC1 signaling. Mol Cell 47:349–58 doi: 10.1016/j.molcel.2012.05.043 22749528

[bib15] Edgar LG McGhee JD 1986 Embryonic expression of a gut-specific esterase in *Caenorhabditis elegans*. Dev Biol 114:109–18 doi: 10.1016/0012-1606(86)90387-8 3956859

[bib16] Edgley ML Baillie DL Riddle DL Rose AM 2006 Genetic balancers. WormBook, ed. The *C. elegans* Research Community, WormBook doi: 10.1895/wormbook.1.89.1

[bib17] Entchev EV Schwudke D Zagoriy V Matyash V Bogdanova A Habermann B 2008 LET-767 is required for the production of branched chain and long chain fatty acids in *Caenorhabditis elegans*. J Biol Chem 283:17550–60 doi: 10.1074/jbc.M800965200 18390550

[bib18] Fielenbach N Antebi A 2008 *C. elegans* dauer formation and the molecular basis of plasticity. Genes Dev 22:2149–65 doi: 10.1101/gad.1701508 18708575 PMC2735354

[bib19] Gems D Sutton AJ Sundermeyer ML Albert PS King KV Edgley ML 1998 Two pleiotropic classes of daf-2 mutation affect larval arrest, adult behavior, reproduction and longevity in *Caenorhabditis elegans*. Genetics 150:129–55 9725835 10.1093/genetics/150.1.129 PMC1460297

[bib20] Gerdt S Lochnit G Dennis RD Geyer R 1997 Isolation and structural analysis of three neutral glycosphingolipids from a mixed population of *Caenorhabditis elegans* (Nematoda:Rhabditida). Glycobiology 7:265–75 doi: 10.1093/glycob/7.2.265 9134433

[bib21] Griffitts JS Huffman DL Whitacre JL Barrows BD Marroquin LD Muller R 2003 Resistance to a bacterial toxin is mediated by removal of a conserved glycosylation pathway required for toxin-host interactions. J Biol Chem 278:45594–602 doi: 10.1074/jbc.M308142200 12944392

[bib22] Han JM Jeong SJ Park MC Kim G Kwon NH Kim HK 2012 Leucyl-tRNA synthetase is an intracellular leucine sensor for the mTORC1-signaling pathway. Cell 149:410–24 doi: 10.1016/j.cell.2012.02.044 22424946

[bib23] Hannun YA Obeid LM 2008 Principles of bioactive lipid signalling: lessons from sphingolipids. Nat Rev Mol Cell Biol 9:139–50 doi: 10.1038/nrm2329 18216770

[bib24] Hansen M Chandra A Mitic LL Onken B Driscoll M Kenyon C 2008 A role for autophagy in the extension of lifespan by dietary restriction in *C. elegans*. PLoS Genet 4:e24 doi: 10.1371/journal.pgen.0040024 18282106 PMC2242811

[bib25] Hansen M Taubert S Crawford D Libina N Lee SJ Kenyon C 2007 Lifespan extension by conditions that inhibit translation in *Caenorhabditis elegans*. Aging Cell 6:95–110 doi: 10.1111/ace.2007.6.issue-1 17266679

[bib26] He C Klionsky DJ 2009 Regulation mechanisms and signaling pathways of autophagy. Annu Rev Genet 43:67–93 doi: 10.1146/annurev-genet-102808-114910 19653858 PMC2831538

[bib27] Hietakangas V Cohen SM 2009 Regulation of tissue growth through nutrient sensing. Annu Rev Genet 43:389–410 doi: 10.1146/annurev-genet-102108-134815 19694515

[bib28] Honjoh S Yamamoto T Uno M Nishida E 2009 Signalling through RHEB-1 mediates intermittent fasting-induced longevity in *C. elegans*. Nature 457:726–30 doi: 10.1038/nature07583 19079239

[bib29] Howell JJ Manning BD 2011 mTOR couples cellular nutrient sensing to organismal metabolic homeostasis. Trends Endocrinol Metab 22:94–102 doi: 10.1016/j.tem.2010.12.003 21269838 PMC3744367

[bib30] Johnson TE Mitchell DH Kline S Kemal R Foy J 1984 Arresting development arrests aging in the nematode *Caenorhabditis elegans*. Mech Ageing Dev 28:23–40 doi: 10.1016/0047-6374(84)90150-7 6542614

[bib31] Jones KT Greer ER Pearce D Ashrafi K 2009 Rictor/TORC2 regulates *Caenorhabditis elegans* fat storage, body size, and development through sgk-1. PLoS Biol 7:e60 doi: 10.1371/journal.pbio.1000060 19260765 PMC2650726

[bib32] Kamath RS Fraser AG Dong Y Poulin G Durbin R Gotta M 2003 Systematic functional analysis of the *Caenorhabditis elegans* genome using RNAi. Nature 421:231–7 doi: 10.1038/nature01278 12529635

[bib33] Kao G Nordenson C Still M Ronnlund A Tuck S Naredi P 2007 ASNA-1 positively regulates insulin secretion in *C. elegans* and mammalian cells. Cell 128:577–87 doi: 10.1016/j.cell.2006.12.031 17289575

[bib34] Karlsson AA 1997 Analysis of intact polar lipids by high-pressure liquid chromatography mass spectrometry/tandem mass spectrometry with use of thermospray or atmospheric pressure ionization. In: Hamilton RJ, editor. In Lipid analysis in oils and fats. Blackie Academics and Professional, London p. 290–316

[bib35] Kim E Goraksha-Hicks P Li L Neufeld TP Guan KL 2008 Regulation of TORC1 by Rag GTPases in nutrient response. Nat Cell Biol 10:935–45 doi: 10.1038/ncb1753 18604198 PMC2711503

[bib36] Kniazeva M Crawford QT Seiber M Wang CY Han M 2004 Monomethyl branched-chain fatty acids play an essential role in *Caenorhabditis elegans* development. PLoS Biol 2:E257 doi: 10.1371/journal.pbio.0020257 15340492 PMC514883

[bib37] Kniazeva M Euler T Han M 2008 A branched-chain fatty acid is involved in post-embryonic growth control in parallel to the insulin receptor pathway and its biosynthesis is feedback-regulated in *C. elegans*. Genes Dev 22:2102–10 doi: 10.1101/gad.1692008 18676815 PMC2492746

[bib38] Kniazeva M Shen H Euler T Wang C Han M 2012 Regulation of maternal phospholipid composition and IP3-dependent embryonic membrane dynamics by a specific fatty acid metabolic event in *C. elegans*. Genes Dev 26:554–66 doi: 10.1101/gad.187054.112 22426533 PMC3315117

[bib39] Laplante M Sabatini DM 2012 mTOR signaling in growth control and disease. Cell 149:274–93 doi: 10.1016/j.cell.2012.03.017 22500797 PMC3331679

[bib40] Lee BH Ashrafi K 2008 A TRPV channel modulates *C. elegans* neurosecretion, larval starvation survival, and adult lifespan. PLoS Genet 4:e1000213 doi: 10.1371/journal.pgen.1000213 18846209 PMC2556084

[bib41] Lemire BD Behrendt M DeCorby A Gaskova D 2009 *C. elegans* longevity pathways converge to decrease mitochondrial membrane potential. Mech Ageing Dev 130:461–5 doi: 10.1016/j.mad.2009.05.001 19442682

[bib42] Li J Wang F Haraldson K Protopopov A Duh FM Geil L 2004 Functional characterization of the candidate tumor suppressor gene NPRL2/G21 located in 3p21.3C. Cancer Res 64:6438–43 doi: 10.1158/0008-5472.CAN-03-3869 15374952

[bib43] Liu JL Desjardins D Branicky R Agellon LB Hekimi S 2012 Mitochondrial oxidative stress alters a pathway in *Caenorhabditis elegans* strongly resembling that of bile acid biosynthesis and secretion in vertebrates. PLoS Genet 8:e1002553 doi: 10.1371/journal.pgen.1002553 22438816 PMC3305355

[bib44] Long X Spycher C Han ZS Rose AM Muller F Avruch J 2002 TOR deficiency in *C. elegans* causes developmental arrest and intestinal atrophy by inhibition of mRNA translation. Curr Biol 12:1448–61 doi: 10.1016/S0960-9822(02)01091-6 12225660

[bib45] Lucanic M Held JM Vantipalli MC Klang IM Graham JB Gibson BW 2011 N-acylethanolamine signalling mediates the effect of diet on lifespan in *Caenorhabditis elegans*. Nature 473:226–9 doi: 10.1038/nature10007 21562563 PMC3093655

[bib46] Ma XM Blenis J 2009 Molecular mechanisms of mTOR-mediated translational control. Nat Rev Mol Cell Biol 10:307–18 doi: 10.1038/nrm2672 19339977

[bib47] Marza E Simonsen KT Faergeman NJ Lesa GM 2009 Expression of ceramide glucosyltransferases, which are essential for glycosphingolipid synthesis, is only required in a small subset of *C. elegans* cells. J Cell Sci 122:822–33 doi: 10.1242/jcs.042754 19240113 PMC2714426

[bib48] Menuz V Howell KS Gentina S Epstein S Riezman I Fornallaz-Mulhauser M 2009 Protection of *C. elegans* from anoxia by HYL-2 ceramide synthase. Science 324:381–4 doi: 10.1126/science.1168532 19372430

[bib49] Merrill AH Jr Schmelz EM Dillehay DL Spiegel S Shayman JA Schroeder JJ 1997 Sphingolipids–the enigmatic lipid class: biochemistry, physiology, and pathophysiology. Toxicol Appl Pharmacol 142:208–25 doi: 10.1006/taap.1996.8029 9007051

[bib50] Morii H Kaneda T 1982 Biosynthesis of branched-chain fatty acids from branched-chain amino acids in subcutaneous tissue of the marine little toothed whale, *Stenella caeruleo-alba*. Comp Biochem Physiol B 71:357–65 doi: 10.1016/0305-0491(82)90395-9 7039950

[bib51] Mukhopadhyay A Tissenbaum HA 2007 Reproduction and longevity: secrets revealed by *C. elegans*. Trends Cell Biol 17:65–71 doi: 10.1016/j.tcb.2006.12.004 17187981

[bib52] Munoz MJ Riddle DL 2003 Positive selection of *Caenorhabditis elegans* mutants with increased stress resistance and longevity. Genetics 163:171–80 12586705 10.1093/genetics/163.1.171 PMC1462431

[bib53] Nada S Hondo A Kasai A Koike M Saito K Uchiyama Y 2009 The novel lipid raft adaptor p18 controls endosome dynamics by anchoring the MEK-ERK pathway to late endosomes. Embo J 28:477–89 doi: 10.1038/emboj.2008.308 19177150 PMC2657578

[bib54] Naka T Fujiwara N Yano I Maeda S Doe M Minamino M 2003 Structural analysis of sphingophospholipids derived from *Sphingobacterium spiritivorum*, the type species of genus Sphingobacterium. Biochim Biophys Acta 1635:83–92 doi: 10.1016/j.bbalip.2003.10.010 14729071

[bib55] Natoli TA Smith LA Rogers KA Wang B Komarnitsky S Budman Y 2010 Inhibition of glucosylceramide accumulation results in effective blockade of polycystic kidney disease in mouse models. Nat Med 16:788–92 doi: 10.1038/nm.2171 20562878 PMC3660226

[bib56] Neklesa TK Davis RW 2009 A genome-wide screen for regulators of TORC1 in response to amino acid starvation reveals a conserved Npr2/3 complex. PLoS Genet 5:e1000515 doi: 10.1371/journal.pgen.1000515 19521502 PMC2686269

[bib57] Nicolaides N Ray T 1965 Skin lipids. 3. Fatty chains in skin lipids. The use of vernix caseosa to differentiate between endogenous and exogenous components in human skin surface lipid. J Am Oil Chem Soc 42:702–7 doi: 10.1007/BF02540043 14343880

[bib58] O’Rourke EJ Soukas AA Carr CE Ruvkun G 2009 *C. elegans* major fats are stored in vesicles distinct from lysosome-related organelles. Cell Metab 10:430–5 doi: 10.1016/j.cmet.2009.10.002 19883620 PMC2921818

[bib59] Oku H Mimura K Tokitsu Y Onaga K Iwasaki H Chinen I 2000 Biased distribution of the branched-chain fatty acids in ceramides of vernix caseosa. Lipids 35:373–81 doi: 10.1007/s11745-000-534-x 10858021

[bib60] Oku H Yagi N Nagata J Chinen I 1994 Precursor role of branched-chain amino acids in the biosynthesis of iso and anteiso fatty acids in rat skin. Biochim Biophys Acta 1214:279–87 doi: 10.1016/0005-2760(94)90074-4 7918610

[bib61] Patterson GI Padgett RW 2000 TGF beta-related pathways. Roles in *Caenorhabditis elegans* development. Trends Genet 16:27–33 doi: 10.1016/S0168-9525(99)01916-2 10637628

[bib62] Pellis-van Berkel W Verheijen MH Cuppen E Asahina M de Rooij J Jansen G 2005 Requirement of the *Caenorhabditis elegans* RapGEF pxf-1 and rap-1 for epithelial integrity. Mol Biol Cell 16:106–16 doi: 10.1091/mbc.E04-06-0492 15525675 PMC539156

[bib63] Polley SR Fay DS 2012 A network of genes antagonistic to the LIN-35 retinoblastoma protein of *C. elegans*. Genetics 191:827–43 doi: 10.1534/genetics.112.140152 22542970 PMC3416014

[bib64] Rabbitts BM Ciotti MK Miller NE Kramer M Lawrenson AL Levitte S 2008 glo-3, a novel *Caenorhabditis elegans* gene, is required for lysosome-related organelle biogenesis. Genetics 180:857–71 doi: 10.1534/genetics.108.093534 18780725 PMC2567386

[bib65] Ran-Ressler RR Devapatla S Lawrence P Brenna JT 2008 Branched chain fatty acids are constituents of the normal healthy newborn gastrointestinal tract. Pediatr Res 64:605–9 doi: 10.1203/PDR.0b013e318184d2e6 18614964 PMC2662770

[bib66] Sancak Y Bar-Peled L Zoncu R Markhard AL Nada S Sabatini DM 2010 Ragulator-Rag complex targets mTORC1 to the lysosomal surface and is necessary for its activation by amino acids. Cell 141:290–303 doi: 10.1016/j.cell.2010.02.024 20381137 PMC3024592

[bib67] Schreiber MA Pierce-Shimomura JT Chan S Parry D McIntire SL 2010 Manipulation of behavioral decline in *Caenorhabditis elegans* with the Rag GTPase raga-1. PLoS Genet 6:e1000972 doi: 10.1371/journal.pgen.1000972 20523893 PMC2877737

[bib68] Schroeder LK Kremer S Kramer MJ Currie E Kwan E Watts JL 2007 Function of the *Caenorhabditis elegans* ABC transporter PGP-2 in the biogenesis of a lysosome-related fat storage organelle. Mol Biol Cell 18:995–1008 doi: 10.1091/mbc.E06-08-0685 17202409 PMC1805080

[bib69] Seamen E Blanchette JM Han M 2009 P-type ATPase TAT-2 negatively regulates monomethyl branched-chain fatty acid mediated function in post-embryonic growth and development in *C. elegans*. PLoS Genet 5:e1000589 doi: 10.1371/journal.pgen.1000589 19662161 PMC2716530

[bib70] Sheaffer KL Updike DL Mango SE 2008 The Target of Rapamycin pathway antagonizes pha-4/FoxA to control development and aging. Curr Biol 18:1355–64 doi: 10.1016/j.cub.2008.07.097 18804378 PMC2615410

[bib71] Sonnichsen B Koski LB Walsh A Marschall P Neumann B Brehm M 2005 Full-genome RNAi profiling of early embryogenesis in *Caenorhabditis elegans*. Nature 434:462–9 doi: 10.1038/nature03353 15791247

[bib72] Soukas AA Kane EA Carr CE Melo JA Ruvkun G 2009 Rictor/TORC2 regulates fat metabolism, feeding, growth, and life span in *Caenorhabditis elegans*. Genes Dev 23:496–511 doi: 10.1101/gad.1775409 19240135 PMC2648650

[bib73] Stiernagle T 2006 Maintenance of *C. elegans*. WormBook, ed. The *C. elegans* Research Community, WormBook doi: 10.1895/wormbook.1.101.1 PMC4781397 18050451

[bib74] Syntichaki P Troulinaki K Tavernarakis N 2007 eIF4E function in somatic cells modulates ageing in *Caenorhabditis elegans*. Nature 445:922–6 doi: 10.1016/j.mad.2007.03.002 17277769

[bib75] van der Poel S Wolthoorn J van den Heuvel D Egmond M Groux-Degroote S Neumann S 2011 Hyperacidification of trans-Golgi network and endo/lysosomes in melanocytes by glucosylceramide-dependent V-ATPase activity. Traffic 12:1634–47 doi: 10.1111/j.1600-0854.2011.01263.x 21810155

[bib76] Vellai T Takacs-Vellai K Zhang Y Kovacs AL Orosz L Muller F 2003 Genetics: influence of TOR kinase on lifespan in *C. elegans*. Nature 426:620 doi: 10.1038/426620a 14668850

[bib77] Wu X Tu BP 2011 Selective regulation of autophagy by the Iml1-Npr2-Npr3 complex in the absence of nitrogen starvation. Mol Biol Cell 22:4124–33 doi: 10.1091/mbc.E11-06-0525 21900499 PMC3204073

[bib78] Yabuuchi E Kaneko T Yano I Moss CW Miyoshi N 1983 Sphingobacterium gen. nov., Sphingobacterium spiritivorum comb. nov., Sphingobacterium multivorum comb. nov., Sphingobacterium mizutae sp. nov., and Flavobacterium indologenes sp. nov.: Glucose-Nonfermenting Gram-Negative Rods in CDC Groups IIK-2 and IIb. Int J Syst Bacteriol 33:580–98 doi: 10.1099/00207713-33-3-580

[bib79] Yamashita T Wada R Sasaki T Deng C Bierfreund U Sandhoff K 1999 A vital role for glycosphingolipid synthesis during development and differentiation. Proc Natl Acad Sci USA 96:9142–7 doi: 10.1073/pnas.96.16.9142 10430909 PMC17746

[bib80] Zhang SO Box AC Xu N Le Men J Yu J Guo F 2010 Genetic and dietary regulation of lipid droplet expansion in *Caenorhabditis elegans*. Proc Natl Acad Sci USA 107:4640–5 doi: 10.1073/pnas.0912308107 20176933 PMC2842062

[bib81] Zoncu R Bar-Peled L Efeyan A Wang S Sancak Y Sabatini DM 2012 mTORC1 senses lysosomal amino acids through an inside-out mechanism that requires the vacuolar H(+)-ATPase. Science 334:678–83 doi: 10.1126/science.1207056 22053050 PMC3211112

[bib82] Zoncu R Efeyan A Sabatini DM 2011 mTOR: from growth signal integration to cancer, diabetes and ageing. Nat Rev Mol Cell Biol 12:21–35 doi: 10.1038/nrm3025 21157483 PMC3390257

