# Supplementary material for: A novel sphingolipid-TORC1 pathway critically promotes postembryonic development in Caenorhabditis elegans
Source: eLife. 2013 May 21;2:e00429. doi: 10.7554/eLife.00429 (PMC3660743; doi:10.7554/eLife.00429)
Supplement: Supplementary file 1. — Detailed description of phenotypes listed in Figure 1B. DOI: http://dx.doi.org/10.7554/eLife.00429.016 [file elife00429s001.doc]

Supplementary File 1

| *C. elegans* strains | Supplement | Phenotype | Supplemental Information |
| --- | --- | --- | --- |
| *elo-5(-)* | Solvent | L1 arrest^b^ | 100% L1 arrest, n=518 |
| *elo-5(-)* | 1mM C17ISO | Adult^b^ | 68.8% reached adulthood, n=316 |
| *elo-5(-)* | 1mM C17ISO-d18:1-Ceramide | L1 arrest | 100% F1 L1 arrest, n=100 |
| *elo-5(-)* | 1mM d16-SPA | L1 arrest^c^ | 100% F1 L1 arrest, n=13 |
| *elo-5(-)* | d17iso-SPA from *S. spirit* sphingolipid^d^ | Adult | 78.4% reached adulthood, n=632 |
| *sptl-1 (RNAi)^e^* | Solvent | L1 arrest^f^ | 99% F1 L1 arrest, n=104 |
| *sptl-1 (RNAi)* | 1mM C17ISO | L1 arrest | 100% F1 L1 arrest, n=157 |
| *sptl-1 (RNAi)* | 1mM d17iso-SPA | Adult | 37% reached adulthood, n=48 |
| *fath-1 (RNAi)* | Solvent | L1 arrest^g^ | 100% F2 L1 arrest, n=200 |
| *fath-1 (RNAi)* | 1mM d17iso-SPA | L1 arrest | 100% F2 L1 arrest, n=200 |
| *cgt-1(-), cgt-3(-)^h^* | 1mM C17ISO | L1 arrest^h^ | 2.4% reached adulthood, n=864 |
| *cgt-1(-), cgt-3(-)* | 1mM d17iso-SPA | L1 arrest | 100% L1 arrest, n=100 |

Detailed description of phenotypes in Figure 1B.

a. All *elo-5(-)* animals mentioned in this table were *elo-5(gk208)* mutants.

b. Also see Figure 2B for quantitative data.

c. Feeding of this LCB may have a toxic effect on *elo-5(-)* larvae.

d. The supplementation of purified d17iso-SPA was ~1mM, but could not be quantified precisely.

e. All RNAi treatments were by feeding, all *sptl-1(RNAi)* experiments were done in an *rrf-3* RNAi sensitive mutant background.

f. Also see (Seamen et al 2009).

g. F1 animals showed larval arrest. Also see Figure S2 for related experiment.

h. The genotype is *cgt-1(tm1027)* fed with *cgt-3(RNAi),* see Figure 2H.
